# Stability trophic cascades in food chains

**DOI:** 10.1098/rsos.180995

**Published:** 2018-11-07

**Authors:** David W. Shanafelt, Michel Loreau

**Affiliations:** 1 Centre for Biodiversity Theory and Modelling, Theoretical and Experimental Ecology Station, CNRS and Paul Sabatier University, 09200 Moulis, France; 2 Université de Lorraine, Université de Strasbourg, AgroParis Tech, Centre National de la Recherche Scientifique, Institut National de la Recherche Agronomique, Bureau d'Economie Théorique et Appliquée, 54000 Nancy, France

**Keywords:** cascade effects, food chain, invariability, stability

## Abstract

While previous studies have evaluated the change in stability for the addition or removal of individual species from trophic food chains and food webs, we know of no study that presents a general theory for how stability changes with the addition or removal of trophic levels. In this study, we present a simple model of a linear food chain and systematically evaluate how stability—measured as invariability—changes with the addition or removal of trophic levels. We identify the presence of trophic cascades in the stability of species. Owing to top-down control by predation and bottom-up regulation by prey, we find that stability of a species is highest when it is at the top of the food chain and lowest when it is just under the top of the food chain. Thus, stability shows patterns identical to those of mean biomass with the addition or removal of trophic levels in food chains. Our results provide a baseline towards a general theory of the effect of adding or removing trophic levels on stability, which can be used to inform empirical studies.

## 1. Introduction

The works of Elton [1], Lindeman [2], May [3], Pimm [4] and others sparked a plethora of research in the field of food webs [5,6], yet we know of no work that systematically evaluates the effect of food chain length on the stability of linear food chains. In this paper, we present a simple model of a linear food chain, illustrate how stability changes with the addition of trophic levels, and identify the presence of trophic cascades in the stability of species.

Since the initial contributions of Elton [1] and Lindeman [2], there have been numerous theoretical and empirical studies investigating the many aspects of food webs including the identification of the structural properties of natural food webs [7], classification of species into trophic levels [8,9], construction of artificial food webs with the properties of real ones [10,11] and quantification of food web structure and complexity [12–15]. The literature on food webs is vast, and we make no attempt to review it in its entirety. Books by Pimm [4], DeAngelis [16], Loreau [5] and McCann [6] provide comprehensive perspectives of the literature, while papers by Paine [17], DeAngelis *et al.* [18], Morin & Lawler [19], Polis & Strong [8], Amarasekare [20], Hall [21], and Brose *et al*. [22] give more focused reviews.

The most relevant part of the food web literature to our work lies in the observations of direct and indirect effects of the change in a species' abundance on the overall food web, often broadly termed ‘cascade effects’ [23–25]. The effect of addition or removal of a species cascades through all levels of the food chain. How a particular species is affected depends on its level in the food chain and the length of the food chain. Growth of species at odd-numbered levels are limited by top-down control by predation; even-numbered species are limited by bottom-up regulation by prey [24,26,27]. This pattern in the distribution of biomass between trophic levels at equilibrium was later extended to consider more complex food web structures [28], convex and logistic growth functions [29] and biodiversity within trophic levels [30]. Empirical evidence suggests that cascade effects are common in nature [31–33], though the strength of the effect is likely to be weaker in terrestrial than in aquatic ecosystems [31].

In our study, we focus on how stability changes with the addition or removal of trophic levels in a food chain. Much of the initial analyses on the stability of food webs centred on the relationship between food web complexity and stability, and the types and strengths of species interactions necessary to achieve it [3,12,34]. Many of these studies considered the stability of a fixed point equilibrium or the probability of encountering such a point, and numerous papers have evaluated the equilibrium dynamics of specific food web structures (see Rosenzweig [35], Hastings & Powell [36], DeAngelis [16] and Post *et al*. [37] for examples of three-species food chains). Recently, scientists have shifted to general measures of stability such as persistence, resilience, resistance and variability [38,39]. While these measures are interrelated [40,41], they capture different perspectives of stability and differ in their applicability to empirical data. While we are aware of other studies that evaluate the change in stability for the addition or removal of an individual species [31,42,43], we know of no general theory for how stability changes with the addition or removal of trophic levels.

**Figure 1.**
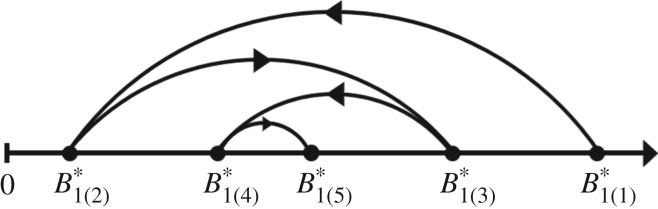
Visual representation of how equilibrium biomass changes with the addition of trophic levels [5]. The notation $B_{i(j)}^{*}$ represents the equilibrium biomass of species *i* for a trophic food chain of length *j*. Results are presented for the basal resource, although the same pattern holds for all species (electronic supplementary material, B).

Beginning with a uni-trophic single species model, we systematically build up to a penta-trophic linear food chain. We evaluate how stability—defined as invariability, the inverse of temporal variability [44–46]—changes with the addition of trophic levels. The most similar studies to ours are the works of Hairston *et al.* [25], Fretwell [26] and Oksanen *et al*. [24], which were generalized by Loreau [5]. They found that equilibrium biomass for each species exhibits a distinct pattern with the addition of trophic levels, which depends on the level of top-down and bottom-up controls. A species possesses its highest equilibrium biomass if it is at the top of the food chain, the lowest when it is just under the top, and a consistent intermediate switching pattern for higher-order food chains (figure 1). However, the focus of these studies—and their subsequent extensions [28–30]—has been solely on patterns in the distribution of equilibrium biomass.

We extend this literature to consider patterns of stability across food chains and in doing so highlight the importance of trophic dynamics on stability. We hypothesize that we will find a similar pattern to equilibrium biomass. That is, top-down control by predation and bottom-up regulation will alter the mean (equilibrium) and temporal standard deviation of biomass to drive the stability of individual species in the food chain. We predict that top-down predation by the top predator will be the most destabilizing, while bottom-up regulation will stabilize the biomass of the top species. Indeed, we find that a species has the highest stability when it is at the top of the food chain (highest equilibrium biomass) and lowest when it is just under the top (lowest equilibrium biomass).

Our results provide a baseline for a general theory for the effect of adding or removing trophic levels on stability, which can be tested in empirical studies.

## 2. Methods

### 2.1. The model

We consider a general food chain model based on the ecosystem models of DeAngelis [16] and Loreau & Holt [47]. By varying the number of trophic levels we study how stability—defined as the ratio of the mean and temporal standard deviation of biomass—changes with the addition or removal of trophic levels.

For a trophic food chain of length *n*, the change in the biomass of each species is given by

2.1 $$\left.\begin{array}{rl} \frac{dB_{1}}{dt}= & I(1+\varepsilon_{I})-lB_{1}-a_{2}B_{1}B_{2}+B_{1}\varepsilon_{1},\\ \frac{dB_{2}}{dt}= & a_{2}b_{2}B_{1}B_{2}-a_{3}B_{2}B_{3}-m_{2}B_{2}+B_{2}\varepsilon_{2},\\ \frac{dB_{3}}{dt}= & a_{3}b_{3}B_{2}B_{3}-a_{4}B_{3}B_{4}-m_{3}B_{3}+B_{3}\varepsilon_{3},\\ \vdots & \\ \;\mathrm{and}\;\frac{dB_{n}}{dt}= & a_{n}b_{n}B_{n-1}B_{n}-m_{n}B_{n}+B_{n}\varepsilon_{n}, \end{array}\right\}$$

where *B*_1_ represents the basal (abiotic) resource, *B*_2_ represents a primary producer (plant), *B*_3_ represents a primary consumer (herbivore) and *B_i_* for *i* > 3 represent secondary consumers (carnivores). The parameters *I* and *ɛ_I_* govern the quantity of resource influx that flows into the community. The parameter *I* is the baseline quantity of resource influx. We introduce stochasticity in the rate of resource influx (growth rate of the resource), where *ɛ_I_* is an independent, normally distributed variable with a mean of zero and variance of *ζ_I_*.^1^ Resources are lost at a constant proportion *l*. The predation rates, biomass conversion efficiencies and mortality rates of consumers are given by *a_i_*, *b_i_* and *m_i_*, respectively.

The final group of terms in (2.1) represents the effect of environmental stochasticity on the growth rates of each species. White noise is applied to each species as an independent, normally distributed random variable *ɛ_i_* with a mean of zero and variances of *ξ_i_*. Each stochastic effect only affects a single trophic level and does not exhibit temporal autocorrelation. We motivate our choice of stochasticity for two reasons. Empirically, we would expect all species to exhibit some degree of temporal variation in their population biomass (*sensu* Schaffer *et al*. [48]), and our formulation is a base case of modelling this. Second, in the absence of noise the system of equations in (2.1) converges to a stable, non-fluctuating equilibrium. In order to measure stability without perturbing the system, we require some sort of fluctuation in biomass around equilibrium [41].

We vary the number of trophic levels in (2.1) to consider uni-trophic (only the basal resource), bi-trophic (basal resource and primary producer), tri-trophic, quadri-trophic and pentra-trophic food chains. The last three food chains include one, two and three consumers, respectively.

### 2.2. Calculating stability

In order to study stability across trophic food chains, we assume an interior solution in which all species coexist—a reasonable assumption when studying linear food chains. We define stability as invariability, the inverse of temporal variability or the ratio of the mean and standard deviation of biomass [44–46]. While other stability measures exist in the literature, invariability is simple to interpret, straightforward to derive analytically and numerically, and can be readily applied to empirical data [44–46]. We derive invariability in two ways. First, we analytically calculate invariability via the Lyapunov equation [49,50]. This requires linearizing the system and studying the dynamics around the equilibrium in response to a slight perturbation. We then calculate invariability numerically by simulating the system of equations of each food chain. While we still restrict our analysis to cases in which all species coexist, the numerical method relaxes the assumption of linearization and allows the system to deviate from the region close to equilibrium.

To analytically calculate invariability, we derive the stationary covariance matrix in the vicinity of the equilibrium population dynamics of each food chain. This tells us how each species responds to stochasticity in its own biomass and the biomass of other species. The system of equations representing the equilibrium (linearized) dynamics of the food chain is given by

2.2 $$\frac{dX}{dt}=AX(t)+E(t),$$

where **X**(*t*) is a vector representing the difference between the states of the system at time *t* and their equilibrium values, **A** is a matrix describing the deterministic dynamics of the system around the equilibria (the Jacobian evaluated at equilibrium) and **E**(*t*) is a vector capturing the effects of stochasticity on the dynamics around the equilibrium. Given the functional forms of stochasticity in (2.1), the vector **E**(*t*) is written explicitly as

2.3 $$E(t)=\left[\begin{array}{ccccc} I & B_{1}^{*} & 0 & 0 & 0\\ 0 & 0 & B_{2}^{*} & 0 & 0\\ 0 & 0 & 0 & \ddots & \vdots \\ 0 & 0 & 0 & \cdots & B_{n}^{*} \end{array}\right]\;\left[\begin{array}{c} \varepsilon_{I}\\ \varepsilon_{1}\\ \vdots \\ \varepsilon_{n} \end{array}\right],$$

where $B_{i}^{*}$ is the equilibrium biomass of species *i*. It is worth recalling that each stochastic event affects each species independently and is not correlated through time.

The state variables of equation (2.2) can be seen as stochastic variables. To calculate the stationary covariance matrix, we evaluate the variance of the left- and right-hand sides of equation (2.2). This collapses to the Lyapunov equation

2.4 $$AC+AC^{T}+MV_{\varepsilon }M^{T}=0,$$

where *A* is defined above, and the matrices *M* and *V*_*ɛ*_ are defined as

2.5 $$M=\left[\begin{array}{ccccc} I & B_{1}^{*} & 0 & 0 & 0\\ 0 & 0 & B_{2}^{*} & 0 & 0\\ 0 & 0 & 0 & \ddots & \vdots \\ 0 & 0 & 0 & \cdots & B_{n}^{*} \end{array}\right]$$

2.6 $$\;\mathrm{and}\;\;V_{\varepsilon }=\left[\begin{array}{cccc} \zeta_{I} & 0 & 0 & 0\\ 0 & \xi_{1} & 0 & 0\\ 0 & 0 & \ddots & \vdots \\ 0 & 0 & \cdots & \xi_{n} \end{array}\right].$$

The matrix *V*_*ɛ*_ is the variance–covariance matrix of stochasticity. The diagonal entries of *V*_*ɛ*_ represent the variances of white noise imposed on the rate of resource influx and on each species (*ζ_I_* and *ξ_i_*, respectively).

The matrix *C* is the stationary covariance matrix that satisfies the Lyapunov equation [41,45,51]. The diagonals of *C* are the variances of the various species around their equilibria given environmental stochasticity. The off-diagonals are the covariances between species, e.g. how the equilibrium biomasses of two species covary around their equilibria in response to a stochastic effect in the other species.

We calculate invariability directly from the matrix *C*. We derive invariability per species as the inverse of the coefficient of variation of biomass for each species, e.g. the mean (equilibrium) biomass of each species divided by its standard deviation of biomass (the square root of the diagonal elements of *C*). Invariability of the entire system is defined as the inverse of the coefficient of variation in the total biomass of all species in the system. An illustration of the Lyapunov method for a bi-trophic system can be found in electronic supplementary material, A.

In addition, we evaluate invariability of each trophic food chain numerically. We simulate the system of nonlinear equations of each trophic food chain in (2.1) using a first-order Euler approximation with step size Δ*t*. Initial conditions are set at the equilibrium values of each species in the absence of stochasticity, and parameter values held constant across food chains. We run each simulation for a time horizon *T* and check that no species went extinct during the simulation. We calculate invariability for each species and total biomass from the final 1000 time steps of each simulation. A full list of parameters is found in table 1.^2^

**Table 1.** Model parameters. The subscript *i* indicates the species or trophic level: basal resource (1), primary producer (2), primary consumer (3), secondary consumer (4) and tertiary consumer (5). For numerical simulations, initial conditions were taken as the equilibrium values in the absence of stochasticity. Parameter values were chosen to guarantee the presence of a stable, interior equilibrium and were held constant across food chains (e.g. *a*_2_ = 0.2 for bi- to penta-trophic food chains). Variances of white noise were equal for all species and were chosen such that stochastic effects neither perturbed the system out of the coexistence basin of attraction nor caused the stochastic additive terms to exceed mortality.^a^

parameter	interpretation	value
*I*	baseline rate of resource influx	variable [5, 150]
*I*	resource loss rate	0.1
*a_i_*	predator predation rate	*a*_2_ = *a*_3_ = *a*_4_ = 0.2 *a*_5_ = 0.5
*b_i_*	biomass conversion efficiency	*b*_2_ = *b*_3_ = 0.3 *b*_4_ = *b*_5_ = 0.4
*m_i_*	mortality rate	0.1
**ɛ*_I_*	white noise (rate of resource influx)	$∼N(0,\zeta_{I})$
**ɛ*_i_*	white noise (per species)	$∼N(0,\xi_{i})$
*ζ_I_*	variance of white noise (rate of resource influx)	0.0100
*ξ_i_*	variance of white noise (per species)	0.0025
*T*	simulation time horizon	1500
Δ*t*	simulation step size	0.1

^a^As pointed out by a reviewer, in order to be a truly self-contained food chain the effect of stochasticity on population biomass should come in the form of mortality only. Indeed, if a positive stochastic effect were to exceed mortality then it would result in a net input or subsidy to biomass.

## 3. Results

### 3.1. Equilibrium biomass, temporal standard deviation in biomass and synchrony between species

We recover the well-established equilibrium biomass results characteristic of top-down predator control in linear food chains [5,24–26]. That is, a species has its highest equilibrium biomass when it is at the top of the food chain, and the lowest when it is at the level below the top (figure 1; electronic supplementary material, B). In the absence of top-down control, species growth is limited solely by the quantity of prey. By contrast, when a species is just below the top of the food chain, it experiences top-down control by an unbounded predator and bottom-up limitation by the availability of its prey. As we increased the number of trophic levels, there is a pattern of intermediate switching of equilibrium biomass for each species.

**Figure 2.**
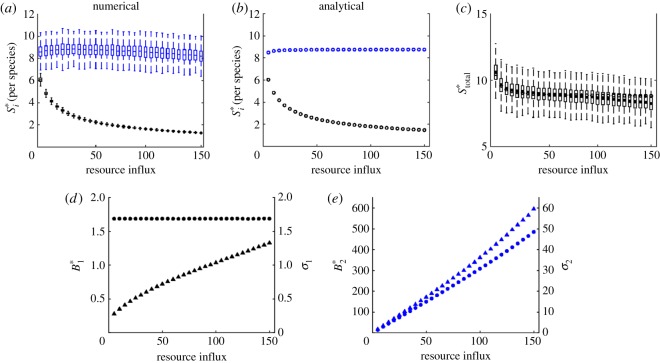
Stability in a *bi-trophic* food chain. (*a*–*c*) Numerical and analytical results for stability per species, and stability of total biomass. Invariability for each species is calculated as the ratio of equilibrium biomass and the standard deviation of biomass (*d*,*e*). Colour and subscript indicate species: basal resource (black, 1) and primary producer (blue, 2). In (*a*,*c*), solid bars indicate the mean values of 1000 simulations. Boxes represent the 25th and 75th percentiles of simulation results. Whiskers correspond to approximately 2.5 standard deviations from the mean. In (*d*,*e*), the marker shape denotes equilibrium biomass (circle) and standard deviations of biomass (triangle). Markers are the mean value of the simulation results.

**Figure 3.**
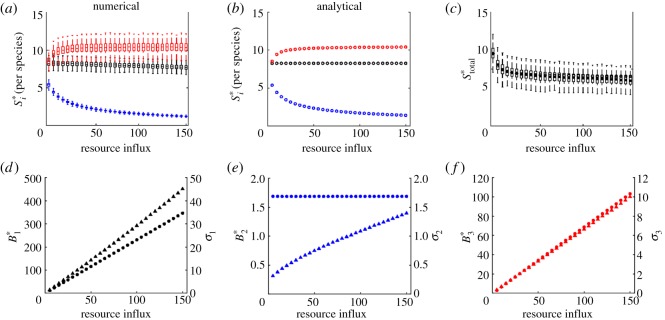
Stability in a *tri-trophic* food chain. (*a*–*c*) Numerical and analytical results for stability per species, and stability of total biomass. Invariability for each species is calculated as the ratio of equilibrium biomass and the standard deviation of biomass (*d*–*f*). Colour and subscript indicate species: basal resource (black, 1), primary producer (blue, 2) and primary consumer (red, 3). In (*a*,*c*), solid bars indicate the mean values of 1000 simulations. Boxes represent the 25th and 75th percentiles of simulation results. Whiskers correspond to approximately 2.5 standard deviations from the mean. In (*d*–*f*) the marker shape denotes equilibrium biomass (circle) and standard deviations of biomass (triangle). Markers are the mean value of the simulation results.

**Figure 4.**
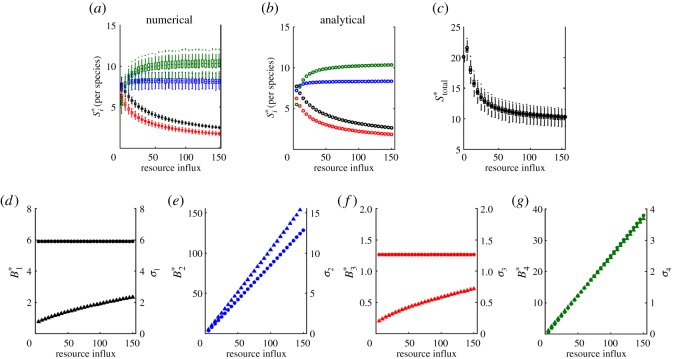
Stability in a *quadri-trophic* food chain. (*a*–*c*) Numerical and analytical results for stability per species, and stability of total biomass. Invariability for each species is calculated as the ratio of equilibrium biomass and the standard deviation of biomass (*d*–*g*). Colour and subscript indicate species: basal resource (black, 1), primary producer (blue, 2), primary consumer (red, 3) and secondary consumer (green, 4). In (*a*,*c*), solid bars indicate the mean values of 1000 simulations. Boxes represent the 25th and 75th percentiles of simulation results. Whiskers correspond to approximately 2.5 s.d. from the mean. In (*d*–*g*), the marker shape denotes equilibrium biomass (circle) and standard deviations of biomass (triangle). Markers are the mean value of the simulation results.

**Figure 5.**
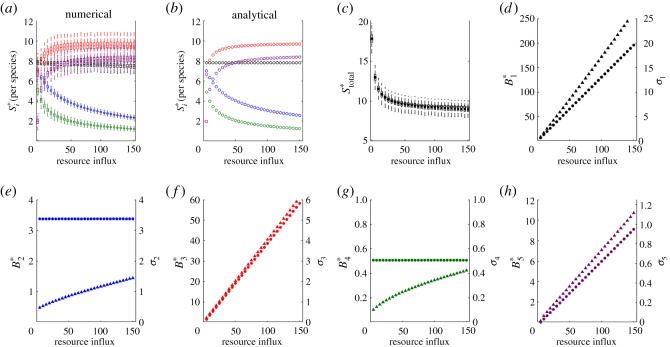
Stability in a *penta-trophic* food chain. (*a*–*c*) Numerical and analytical results for stability per species, and stability of total biomass. Invariability for each species is calculated as the ratio of equilibrium biomass and the standard deviation of biomass (*d*–*h*). Colour and subscript indicate species: basal resource (black, 1), primary producer (blue, 2), primary consumer (red, 3), secondary consumer (green, 4) and tertiary consumer (purple, 5). In (*a*,*c*), solid bars indicate the mean values of 1000 simulations. Boxes represent the 25th and 75th percentiles of simulation results. Whiskers correspond to approximately 2.5 s.d. from the mean. In (*d*–*h*), marker shape denotes equilibrium biomass (circle) and standard deviations of biomass (triangle). Markers are the mean value of the simulation results.

In addition, we recover the well-known pattern in the response of equilibrium biomass to nutrient enrichment (figures 2–5) [24,26]. For all food chains, total biomass increases with nutrient enrichment. However, the equilibrium biomass of each species depends on its place in the food chain. If the number of trophic levels is odd, then equilibrium biomass increases with the rate of resource influx in odd-numbered trophic levels and is constant in even-numbered ones. If the number of trophic levels is even, then equilibrium biomass increases with resource influx in even-numbered levels and is constant in odd-numbered ones. Consider, for example, a trophic food chain with *n* levels. The *n*th level consumer (unbounded top predator) exerts top-down control on the *n* − 1 level consumer, which relieves predation pressure on the *n* − 2 consumer. The *n* − 2 consumer then exerts top-down control on the *n* − 3 level, which relieves predation pressure on the *n* − 4 level (and so on).

We find the same patterns in the temporal standard deviations in biomass for each species. A species has the highest standard deviation in biomass if it is at the top of the food chain, lowest if it is just under the top, and a pattern of switching between intermediate trophic levels (electronic supplementary material, B). For odd-numbered (even-numbered) species, the temporal standard deviation in biomass increases with the baseline rate of resource influx when the number of trophic levels is odd (even), and increases at a lower rate when the number of trophic levels is even (odd). As we will see below, these results have implications on stability.

Species covariances—calculated from the off-diagonals of the stationary covariance matrix—are consistent with our findings of equilibrium biomass and the temporal standard deviations in biomass (electronic supplementary material, B). How the biomass of one species fluctuates around its equilibrium in response to a perturbation in the biomass of another species depends on the trophic level of each species and the length of the food chain. The covariance between a predator and its prey is always negative. The cascade effects described above govern the covariances of the remaining species. In bi-, tri- and quadri-trophic food chains the covariances between two odd-numbered (or two even-numbered) species is positive, while the covariances between an odd-numbered and an even-numbered species are negative. The covariances of species in a penta-trophic food chain are generally consistent with this, though there is more nonlinearity in the response of the system to increases in the baseline rate of resource influx.

### 3.2. Stability within individual food chains

Our results from bi-trophic to penta-trophic food chains are presented in figures 2–5. We find a general agreement between analytical predictions derived from the linear approximation and numerical simulations of the full nonlinear system.

In a bi-trophic food chain, predation by the primary producer destabilizes the biomass of the basal resource (figure 2*a*–*c*). Equilibrium biomass of the basal resource remains constant as the baseline level of resource influx increases. Any new biomass is consumed by the primary producer, whose equilibrium biomass increases with the baseline level of resource influx. The temporal standard deviations in biomass of the two species increase with the baseline resource influx rate (figure 2*d*,*e*). However, the standard deviation in biomass for the primary producer increases at approximately the same rate as equilibrium biomass, causing stability to remain more or less constant.

For tri-, quadri- and penta-trophic food chains, stability patterns are more complicated. At low baseline levels of resource influx, all species coexist but the resource limits the biomass of the higher trophic levels. The top predator exists but at low biomass and low stability. Additional nutrients provide more energy to the system that allow additional biomass and stabilize the system. However, as the baseline rate of resource influx increases and the equilibrium biomass of each species follows, we observe greater exploitation of prey by the top predator. Stability of the species just under the top of the food chain declines, as does the stability of total biomass. Indeed, these results generalize the paradox of enrichment to dynamics in the vicinity of a stable equilibrium [52].

In all food chains, however, the species just below the top of the food chain is the least stable (figures 3–5). The species just below the top of the food chain and those at alternating levels below it experience both top-down control and bottom-up regulation. At equilibrium, these species experience pure top-down control; in the vicinity of its equilibrium, these species experience fluctuations due to bottom-up processes. But in contrast to other species that experience similar top-down control and bottom-up regulation, the species just below the top of the food chain is consumed by an unbounded predator. Biomass of the top predator is regulated solely by the availability of its prey, and the top predator consumes any new biomass of the species just below the top of the food chain.

The lack of top-down control on the biomass of the top predator is the reason that predation by the top predator is more destabilizing than the top-down control of another species. Focusing on the quadri- and penta-trophic food chains, as we increase the baseline rate of resource influx we find that the equilibrium biomass of the species just below the top of the food chain and that of the second trophic level below it remain constant while their standard deviations in biomass increase (figures 4 and 5; electronic supplementary material, B). While the latter species has a higher standard deviation in biomass, the equilibrium biomass of the species just under the top is lower due to predation by the unbounded top predator, resulting in a lower stability. Our results hold when varying other model parameters, such as the rate of predation by the primary producer (electronic supplementary material, B).

### 3.3. Stability across food chains

**Figure 6.**
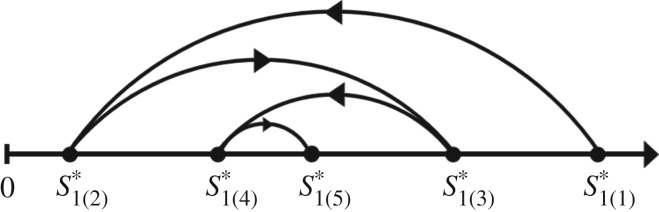
Visual representation of how stability changes with the addition of trophic levels. The notation $S_{i(j)}^{*}$ represents the stability of species *i* for a trophic food chain of length *j*. Results are presented for the basal resource, although we observe the same pattern for all species (electronic supplementary material, B).

Like our results on equilibrium biomass, we find a consistent pattern in the stability of species across food chains (figure 6; electronic supplementary material, B). Species are most stable when they are at the top of the food chain, and least stable when they are just below it. When species are at intermediate positions within food chains, we find a switching pattern of stability. Bottom-up regulation of growth provides a stabilizing effect for the top predator. Top-down control by an unbounded top predator is most destabilizing. At intermediate positions within the food chain, the stability of a species depends on top-down control caused by the cascade effects of the top predator. In even-numbered (odd-numbered) food chains, the top predator relieves predation pressure on other even-numbered (odd-numbered) species and the equilibrium biomasses and standard deviations in biomass increase with the rate of resource influx. At the same time, any new biomass of odd-numbered (even-numbered) species is consumed by even-numbered (odd-numbered) species. Equilibrium biomasses of odd-numbered (even-numbered) species remain constant with the rate of resource influx while the standard deviations in biomass increase. Stability of odd-numbered (even-numbered) species declines, scaling with the level of equilibrium biomass.

Thus we can summarize our findings by the inequalities

3.1 $$\left.\begin{array}{lll} S_{1(2)}^{*}<S_{1(4)}^{*}<S_{1(5)}^{*}<S_{1(3)}^{*}<S_{1(1)}^{*}\; & \; & (\mathrm{basal}\;\mathrm{resource})\;\\ S_{2(3)}^{*}<S_{2(5)}^{*}<S_{2(4)}^{*}<S_{2(2)}^{*}\; & \; & (\mathrm{primary}\;\mathrm{producer})\;\\ S_{3(4)}^{*}<S_{3(5)}^{*}<S_{3(3)}^{*}\; & \; & (\mathrm{primary}\;\mathrm{consumer})\;\\ S_{4(5)}^{*}<S_{4(4)}^{*}\; & \; & (\mathrm{secondary}\;\mathrm{consumer}), \end{array}\right\}$$

where $S_{i(j)}^{*}$ is the stability of species *i* in trophic network *j* (figure 6).

The observed pattern of stability is driven by the interplay between bottom-up regulation of prey and top-down control by predation, which affect the equilibrium biomass and temporal standard deviation in biomass of each species in our food chains. Stability (as invariability) is calculated as the ratio of equilibrium biomass to the temporal standard deviation in biomass. If equilibrium biomass scales at the same rate as the standard deviation in biomass, then stability is insensitive to the baseline rate of resource influx. If equilibrium biomass remains constant while the standard deviation in biomass increases, then stability declines.

## 4. Discussion

Our results for mean biomass and stability within single food chains are generally consistent with existing theory [4,16,35,36] and empirical data [32,53]. For mean biomass, we recover the consistent ‘alternation of regulation’ in equilibrium biomass characteristic of top-down control by predation in linear trophic food chains [24–26]. We also capture the effects of bottom-up regulation due to nutrient or prey limitation [8,16].

For stability, we identify cascade effects due to the addition of novel predators in the food chain. Like the pattern for mean biomass, we find a general pattern in the stability of each species as we add trophic levels. We show that the stability of a species is highest when it is at the top of the food chain, lowest when it is just below the top level, and exhibits a switching pattern at intermediate levels. While previous research has looked at the equilibrium biomass [5,24], the stability of specific food web structures [16,20] and secondary extinctions associated with the removal of species [42,54–56], we provide a first step towards a general theory on how the stability of species changes with the addition of trophic levels. Further, we generalize the paradox of enrichment to the vicinity of stable equilibria. The paradox of enrichment was established for locally unstable systems, e.g. the emergence of limit cycles in response to the addition of nutrients [52]. By analysing dynamics around stable equilibria, we demonstrate the destabilizing effect of nutrient addition and extend the paradox of enrichment to locally stable systems.

Our results have implications outside the food web literature. We find an overall destabilizing effect of resource enrichment, increasing the variability of total biomass in all food chains and of the species just below the top in the tri- to penta-trophic chains. This relates to the literature on the effects of nutrient addition on natural systems. In general, there is evidence that nutrient loading can be destabilizing [16,52,57,58] and the literature on allochthonous inputs suggests that additional resources can be stabilizing or destabilizing, depending on the species affected and preference for resources [8,16,59]. In aquatic systems, the effects of nutrient addition—primarily from terrestrial inflows—and eventual eutrophication of aquatic ecosystems are well documented [60–62]. Empirical evidence suggests the presence of tipping points in the quantity of nutrient loads prior to the transition to a eutrophic state [63]. In our study, extinctions are not possible (by assumption), but our results indicate regions of the parameter space where further nutrient additions will push the system out of the coexistence basin of attraction (electronic supplementary material, B). We further observe differential effects of nutrient loading for each trophic level and nonlinear declines in the stability of total biomass with the addition of nutrients. This suggests that not only is nutrient loading destabilizing, but that this destabilization manifests itself differently depending on the trophic level and structure of the food chain. Nutrient additions enter the food chain via the basal resource, the effects of which then filter up the food chain based on the trophic dynamics in play at each trophic level. Depending on the trophic level and length of the food chain, the means (equilibrium) and temporal standard deviations in biomass can change in different ways in response to nutrient loading.

While we present a general theory for the addition of trophic levels on stability, we do so for simple linear food chains. Many extensions are beyond the scope of our analysis. For example, it would be useful to extend these results to more complex species interactions and food web structures. Though we present our results for type I functional responses, we would expect our conclusions to hold for more complex predator response functions. We present numerical results for a type II functional response in electronic supplementary material, B. Like the type I case, we restrict our analysis to scenarios in which all species coexist. In general, we find the same qualitative results, though there is a larger degree of variation in stability associated with the addition of trophic levels. Oscillatory behaviour destabilizes the system, with species more likely to go extinct due to environmental stochasticity during periods of low population biomass. Other types of interactions and food web structures could include mutualistic interactions [64,65], omnivory and multiple prey species [59,66], intraguild predation [67], parasitism [68] and cannibalism [6,16,20]. Similarly, topological properties of the trophic network such as looping [69] and modularity [70] affect the presence of locally stable equilibria, and spatial structure can alter the dynamics of food webs compared with single-patch systems [20,71]. Indeed, Jager & Gardner [28] and Abrams [30] demonstrated that the ‘alternation of regulation’ pattern in equilibrium biomass does not always hold in more complex food webs, and it would be useful to test how more complex dynamics alter patterns in stability.

We model stochasticity as environmental stochasticity. It would be interesting to model other types of stochasticity—such as demographic stochasticity—though we would expect this to matter most when species populations are small [72]. Similarly, we take stochasticity as a series of independent events uncorrelated across species and time. Other possible extensions include relaxing this assumption by varying the relative strength of stochastic effects across trophic levels or having stochastic effects be correlated between species and time. Environmental shocks—natural or anthropogenic—often affect multiple species regardless of their place in the food chain [73–75], and preliminary results suggest that varying the relative strength of stochastic effects across trophic levels can change stability, though in general the pattern is consistent with our observations [76]. For example, one could envision scenarios where top predators are more sensitive than others to disturbance or perturbation or have greater restrictions on their growth [77,78]. We test the robustness of our results to the top predator possessing higher levels of stochasticity (relative to other species) and density-dependent mortality (electronic supplementary material, B). In each case, we find that our pattern of stability generally holds, particularly for higher trophic levels. When patterns deviate, they do so in a way consistent with our intuition of how stability cascades down the food chain. We leave a detailed analysis of these extensions for future work.

It is possible to test the predictions of our model empirically, such as with micro- or mesocosm experimental set-ups [56,79,80]. In these cases, researchers will require model species with low enough generation times for the system to reach equilibrium and be able to exert enough control on the system to enforce a strict food chain interaction structure (e.g. no omnivory). To calculate stability as invariability, researchers would need to gather data on population abundances (biomass or density) over time [38,46,81].

## Supplementary Material

Supplemental materials A - D

## Footnotes

1 In effect, stochasticity in the rate of resource influx acts as a bottom-up regulation in the growth rate of the resource. In the absence of stochasticity, the growth (influx) rate of the basal resource is taken as constant, which has implications for the pattern of stability of the basal resource across food chains (electronic supplementary material, B).

2 Analytical solutions for invariability were calculated in Wolfram Mathematica 10.2. Numerical simulations were conducted in Matlab 2016. Source code for the analytical and numerical solutions can be found in the electronic supplementary material, C and D, or on the Open Science Framework (https://osf.io/hxfd2/).

## Data accessibility

We include source code for the analytical and numerical solutions in the electronic supplementary material, C and D. Alternatively, all source code and model results can be retrieved from the Open Science Framework (https://osf.io/hxfd2/).
