# Supplementary material for: Stability trophic cascades in food chains
Source: R Soc Open Sci. 2018 Nov 7;5(11):180995. doi: 10.1098/rsos.180995 (PMC6281913; doi:10.1098/rsos.180995)
Supplement: Supplemental materials A - D [file rsos180995supp1.docx]

*Stability trophic cascades in food chains*

Electronic Supplemental Material

David W Shanafelt^1, 2^

david.shanafelt@sete.cnrs.fr

Michel Loreau^1^

michel.loreau@sete.cnrs.fr

^1^Centre for Biodiversity Theory and Modelling

Theoretical and Experimental Ecology Station

CNRS and Paul Sabatier University

09200 Moulis, France

^2^Université de Lorraine

Université de Strasbourg

AgroParis Tech

Centre National de la Recherche Scientifique

Institut National de la Recherche Agronomique

Bureau d'Economie Théorique et Appliquée

54000 Nancy, France

Corresponding author:

David W Shanafelt

Centre for Biodiversity Theory and Modelling

Theoretical and Experimental Ecology Station

CNRS and Paul Sabatier University

09200 Moulis, France

+33 (0) 5 61 04 03 60

david.shanafelt@sete.cnrs.fr

SUPPLEMENTAL MATERIAL A. ILLUSTRATION OF THE LYAPUNOV METHOD FOR A BI-TROPHIC FOOD CHAIN

Consider a general two species, bi-trophic food chain model with a basal resource ($B_{1}$) and primary producer ($B_{2}$) based on the work of [DeAngelis (1992](#_ENREF_2)) and [Loreau and Holt (2004](#_ENREF_3)). The change in biomass of each species at time $t$ is given by the following differential equations:

$$\begin{matrix} \frac{dB_{1}}{dt}=I(1+\varepsilon_{I})-lB_{1}(t)-a_{2}B_{1}(t)B_{2}(t)-B_{1}(t)\varepsilon_{1}(t) \\ \frac{dB_{2}}{dt}=a_{2}b_{2}B_{1}(t)B_{2}(t)-m_{P}B_{2}(t)-B_{2}(t)\varepsilon_{2}(t) \end{matrix}$$

where $I$is the baseline quantity of resource influx that flows into the community. We introduce environmental stochasticity in the rate of resource influx (growth rate), where $\varepsilon_{I}$ is an independent, normally-distributed variable with a mean of zero and variance of $\zeta_{I}$. Resources are lost at a constant proportion $l$. The predation rate of the primary producer on the basal resource is given by $a_{2}$. The primary producer converts resources into new biomass at a constant efficiency $b_{2}$. Mortality rate for the primary producer is given by $m_{2}$. The final group of terms in each equation represents the effect of environmental stochasticity on the growth rates of the resource and primary producer. White noise is applied to each species as independent, normally-distributed random variables ($\varepsilon_{1}$ and $\varepsilon_{2}$) with a mean of zero and variances of $\varsigma_{1}$ and $\varsigma_{2}$. Note that each stochastic effect only affects a single species and does not exhibit temporal autocorrelation.

*Analytical derivation of stability*

In order to calculate invariability, we derive the stationary covariance matrix of the equilibrium (linearized) population dynamics of ([Nisbet and Gurney, 1982](#_ENREF_4); [van Kampen, 1992](#_ENREF_5)). First, we calculate the equilibrium populations of the resource and primary producer in the absence of environmental stochasticity and evaluate the Jacobian at the equilibrium values. The equilibria for the system and the associated Jacobian ($J$) are given by,

Linearizing equation around , we may write the following system of equations to describe the behavior of the system in the vicinity of its equilibria:

$$\frac{dX}{dt}=AX(t)+E(t)$$

where $X(t)$ is a vector representing the difference between the states of the system at time $t$ and their equilibrium values, $A$ is a matrix describing the deterministic dynamics of the system around the equilibria (the Jacobian evaluated at equilibrium), and $E(t)$is a vector capturing the effect of environmental stochasticity. Specifically, we define

Given the functional form of environmental stochasticity in , the function $E(t)$ is written explicitly as,

$$E(t)=\left[\begin{matrix} I & B_{1}^{*} & 0 \\ 0 & 0 & B_{2}^{*} \end{matrix}\right]\left[\begin{matrix} \varepsilon_{I} \\ \varepsilon_{1} \\ \varepsilon_{2} \end{matrix}\right]$$

After matrix multiplication, the resulting matrix in is the effect of stochasticity on the dynamics around the equilibrium. It is worth recalling that each stochastic event affects each species separately (as indicated by their subscripts) and are not correlated with each other or through time.

The state variables in equation can be seen as random variables. To calculate the stationary covariance matrix, we evaluate the variance of the left- and right-hand sides of equation . It can be shown that the stationary covariance matrix ($C$) is the matrix that satisfies the Lyapunov equation ([Arnoldi et al., 2016](#_ENREF_1); [Wang and Loreau, 2016](#_ENREF_6); [Wang et al., 2015](#_ENREF_7)),

$$AC+CA^{T}+MV_{\varepsilon}M^{T}=0$$

where $A$ is defined as above. The matrices $M$ and $V_{\varepsilon}$ are given by,

The matrix $V_{\varepsilon}$ is the variance-covariance matrix of stochasticity. The diagonal entries of $V_{\varepsilon}$ represent the variances of white noise imposed on the rate of resource influx and on each species ($\varsigma_{I}$ and $\varsigma_{i}$ respectively).

The resulting stationary covariance matrix of is given by,

$$C^{*}=\left[\begin{matrix} \frac{m_{2}\left(Ia_{2}b_{2}\left(Ia_{2}b_{2}\varsigma_{I}+\varsigma_{2}\right)+m_{2}^{2}\varsigma_{1}-lm_{2}\varsigma_{2}\right)}{2Ia_{2}^{3}b_{2}^{3}} & \frac{\varsigma_{2}\left(lm_{2}-Ia_{2}b_{2}\right)}{2a_{2}^{2}b_{2}m_{2}} \\ & \\ \frac{\varsigma_{2}\left(lm_{2}-Ia_{2}b_{2}\right)}{2a_{2}^{2}b_{2}m_{2}} & \frac{Ia_{2}b_{2}-lm_{2}}{2Im_{2}^{3}a_{2}^{3}b_{2}}\left(m_{2}^{3}\left(m_{2}\varsigma_{1}-l\varsigma_{2}\right)\right)+ \\ & \frac{Ia_{2}b_{2}-lm_{2}}{2Im_{2}^{3}a_{2}^{3}b_{2}}\left(Im_{2}^{2}\varsigma_{2}a_{2}b_{2}+I^{2}\left(\varsigma_{2}+m_{2}^{2}\varsigma_{I}\right)a_{2}^{2}b_{2}^{2}\right) \end{matrix}\right]$$

The diagonals of $C^{*}$ are the variances of the basal resource and primary producer around their equilibria given environmental stochasticity. The off-diagonals are the covariances between species, e. g. how the equilibrium biomass of one species fluctuates around its equilibrium in response to a stochastic effect in another species.

We calculate invariability directly from the matrix $C^{*}$. We define invariability per species as the inverse of the coefficient of variation of biomass, e.g. the mean biomass from divided by the standard deviation of biomass from ($S_{1}^{*}$and $S_{2}^{*}$ respectively). Invariability in total biomass ($S_{TOTAL}^{*}$) is the inverse of the coefficient of variation in the total biomass of all species in the system. Formally, let

REFERENCES

Arnoldi, J.-F., Loreau, M., and Haegeman, B., 2016. Resilience, reactivity and variability: A mathematical comparison of ecological stability measures. Journal of Theoretical Biology 389, 47-59.

DeAngelis, D.L., 1992. Dynamics of nutrientt cycling and food webs. Chapman and Hall, London.

Loreau, M., and Holt, R.D., 2004. Spatial flows and the regulation of ecosystems. The American Naturalist 163, 606-615.

Nisbet, R.M., and Gurney, W.S.C., 1982. Modelling fluctuating populations. John Wiley & Sons, Chichester.

van Kampen, N.G., 1992. Stochastic processes in physics and chemistry. Elsevier BV, Amsterdam.

Wang, S., and Loreau, M., 2016. Biodiversity and ecosystem stability across scales in metacommunities. Ecology Letters 19, 510-518.

Wang, S., Haegeman, B., and Loreau, M., 2015. Dispersal and metapopulation stability. PeerJ 3, e1295.

SUPPLEMENTAL MATERIAL B. ADDITIONAL TABLES, FIGURES, AND RESULTS

Table S1. List of tables and figures.

| Title |
| --- |
| Figure S1. Equilibrium biomass across food chains (per species) |
| Figure S2. Temporal standard deviation in biomass across food chains  (per species) |
| Figure S3. Stability across food chains (per species) |
|  |
| Figure S4. Equilibrium biomass of each species per food chain |
| Figure S5. Temporal standard deviation in biomass of each species per  food chain |
|  |
| Figure S6. Synchrony between species in bi- and tri-trophic food chains |
| Figure S7. Synchrony between species in a quadri-trophic food chain |
| Figure S8. Synchrony between species in a penta-trophic food chain |
|  |
| Figure S9. Stability of total biomass across food chains |
|  |
| Figure S10. Stability across food chains, varying predation rate of the primary consumer |
|  |
| Figure S11. Stability in a bi-trophic food chain with a type-II functional  response |
| Figure S12. Stability in a tri-trophic food chain with a type-II functional  response |
| Figure S13. Stability across food chains with a type-II functional response |
|  |
| Figure S14-S16. Stability across food chains with a higher rate of  stochasticity in the top predator |
| Figure S17. Stability across food chains with density-dependent mortality  in the top predator |
|  |
| Figure S18. Stability across food chains (per species) in the absence of  stochasticity in the baseline rate of resource influx |
|  |


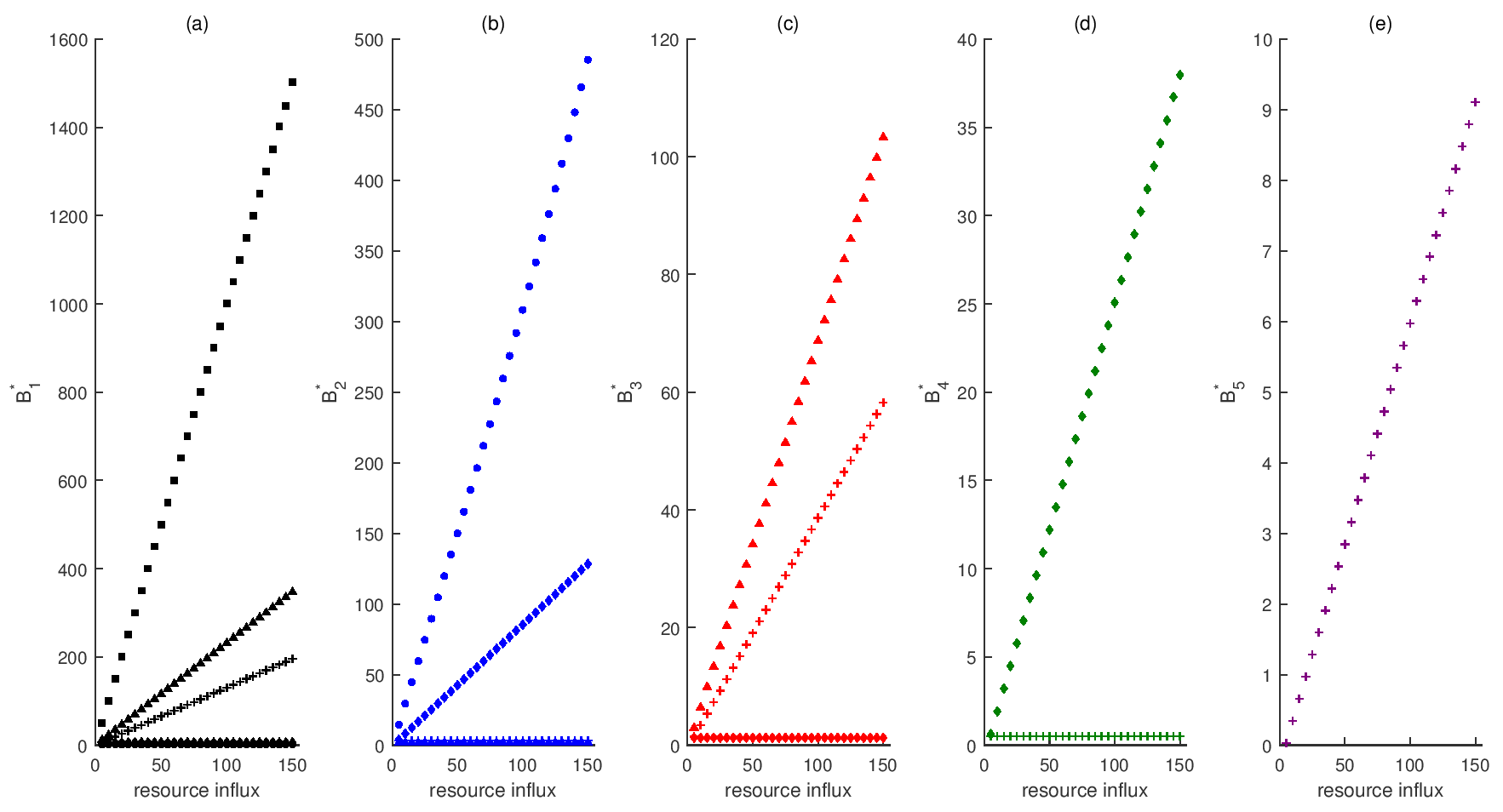


Figure S1. Equilibrium biomass across food chains. Panels and marker colors correspond to the equilibrium biomass of each species: basal resource (a, black), primary producer (b, blue), primary consumer (c, red), secondary consumer (d, green), and tertiary consumer (e, purple). Marker shape denotes the trophic food chain: single species (square), bi-trophic (circle), tri-trophic (triangle), quadri-trophic (diamond), and penta-trophic (plus). Markers are the mean results of 1000 simulations.


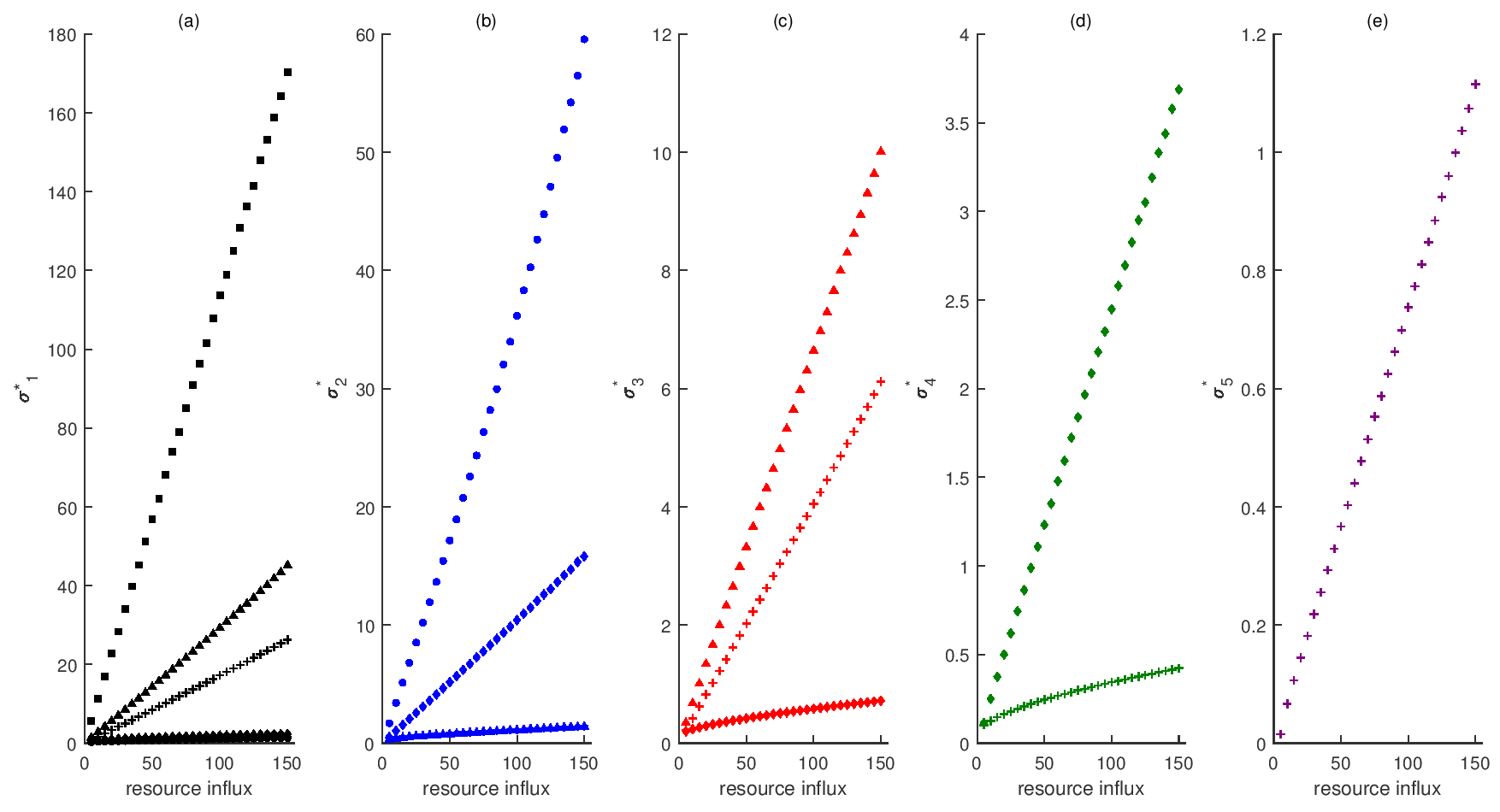


Figure S2. Temporal standard deviation in biomass across food chains. Panels and marker colors correspond to the standard deviation in biomass of each species: basal resource (a, black), primary producer (b, blue), primary consumer (c, red), secondary consumer (d, green), and tertiary consumer (e, purple). Marker shape denotes the trophic food chain: single species (square), bi-trophic (circle), tri-trophic (triangle), quadri-trophic (diamond), and penta-trophic (plus). Markers are the mean results of 1000 simulations.


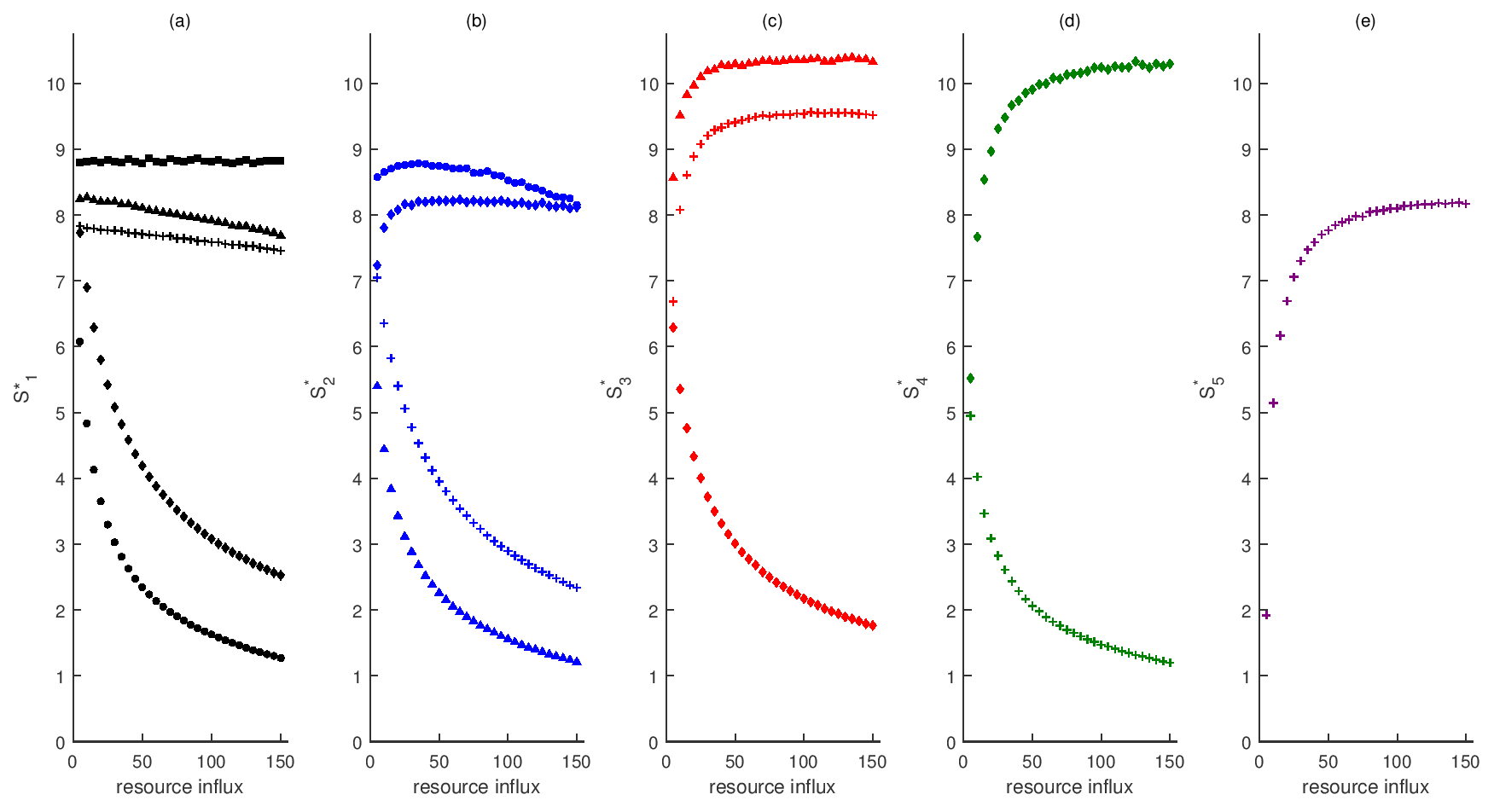


Figure S3. Stability across food chains. Invariability is calculated as the ratio of equilibrium biomass and the standard deviation of biomass (Figures S2 and S3). Panels and marker colors correspond to the stability of species: basal resource (a, black), primary producer (b, blue), primary consumer (c, red), secondary consumer (d, green), and tertiary consumer (e, purple). Marker shape denotes the trophic food chain: single species (square), bi-trophic (circle), tri-trophic (triangle), quadri-trophic (diamond), and penta-trophic (plus). Markers are the mean results of 1000 simulations.


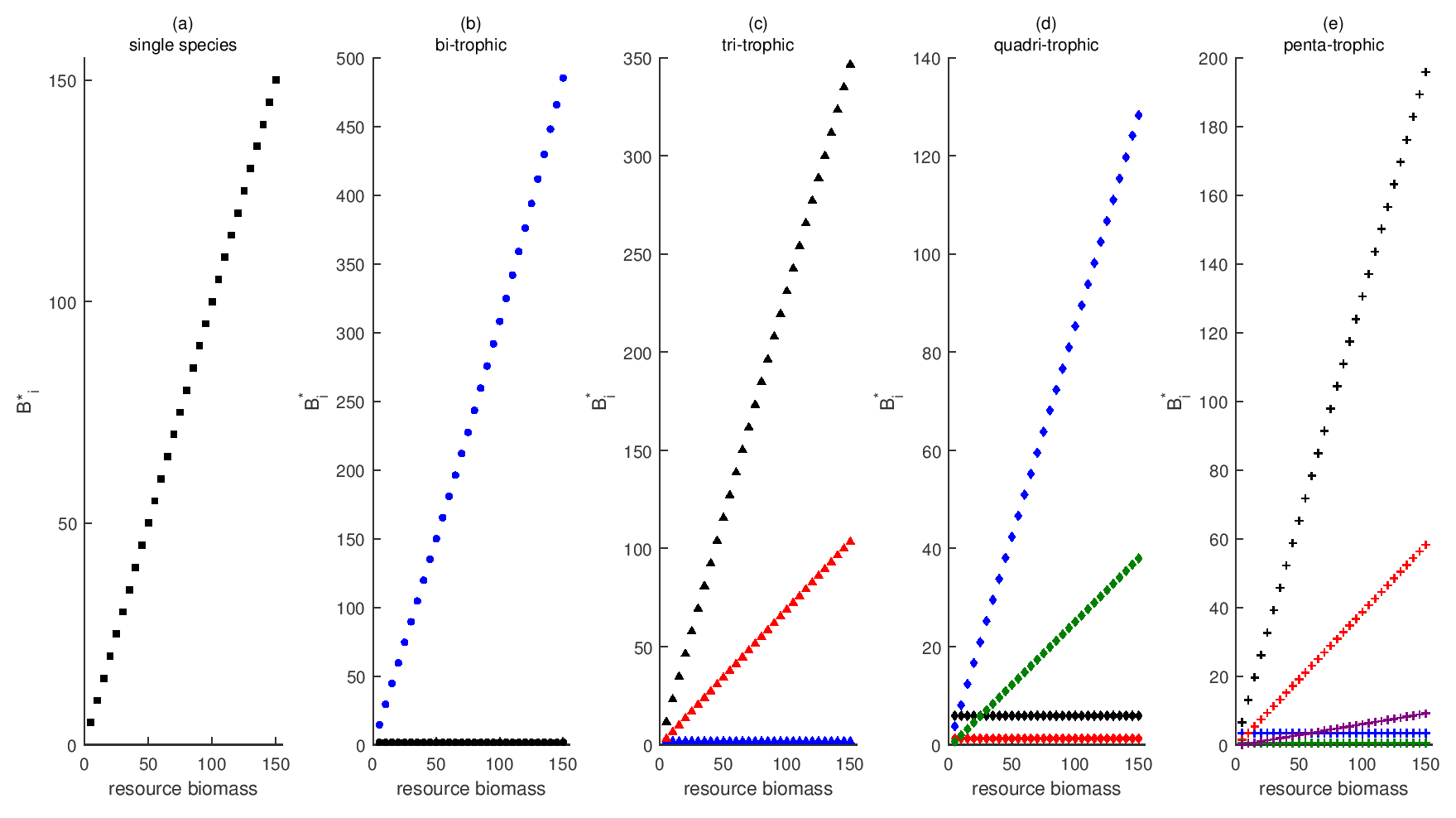


Figure S4. Equilibrium biomass of each species per food chain. Panels and marker shapes denote the trophic food chain: single species (a, square), bi-trophic (b, circle), tri-trophic (c, triangle), quadri-trophic (d, diamond), and penta-trophic (e, plus). Marker colors correspond to the equilibrium biomass of each species: basal resource (black), primary producer (blue), primary consumer (red), secondary consumer (green), and tertiary consumer (purple). Markers are the mean results of 1000 simulations.


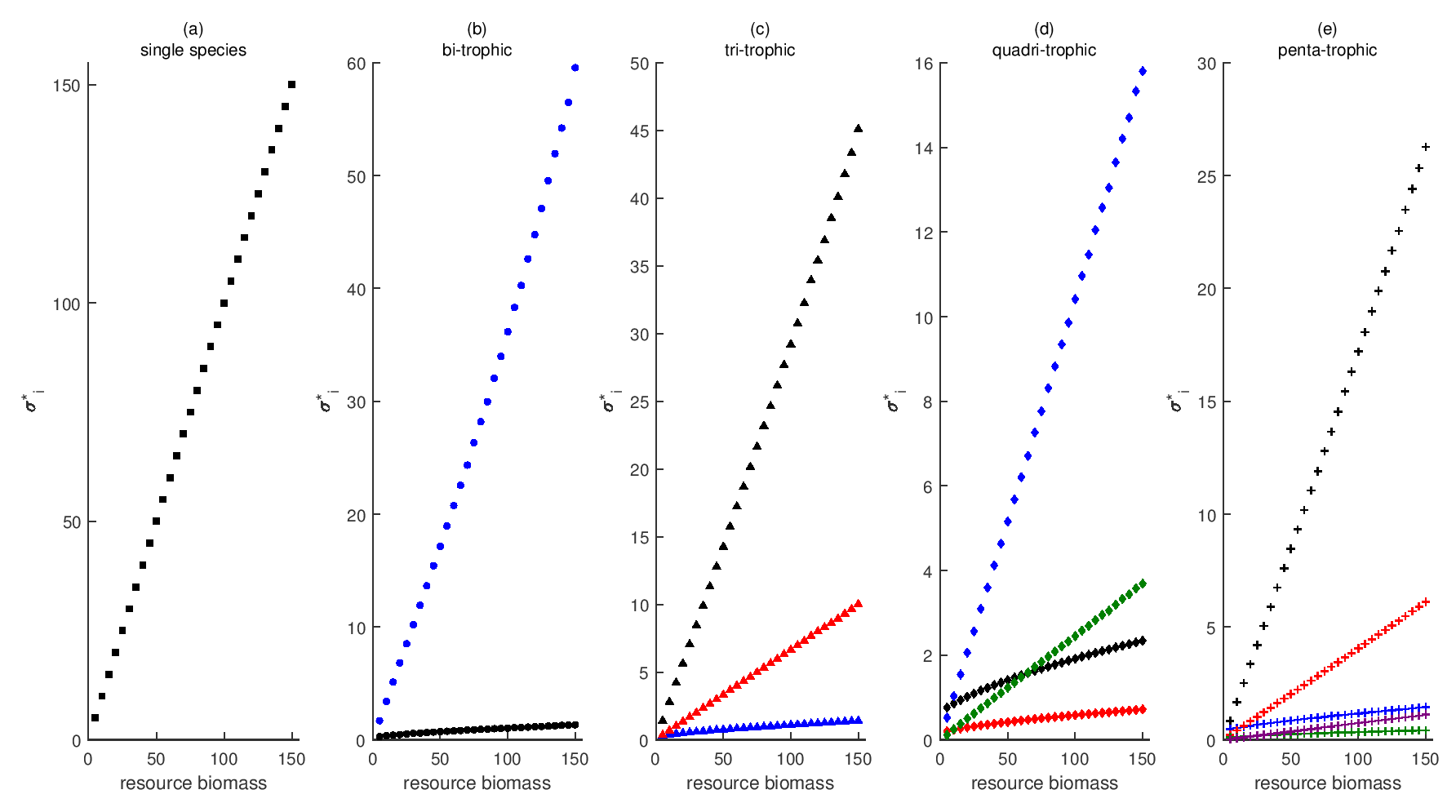


Figure S5. Temporal standard deviation in biomass of each species per food chain. Panels and marker shapes denote the trophic food chain: single species (a, square), bi-trophic (b, circle), tri-trophic (c, triangle), quadri-trophic (d, diamond), and penta-trophic (e, plus). Marker colors correspond to the standard deviation in biomass of each species: basal resource (black), primary producer (blue), primary consumer (red), secondary consumer (green), and tertiary consumer (purple). Markers are the mean results of 1000 simulations.


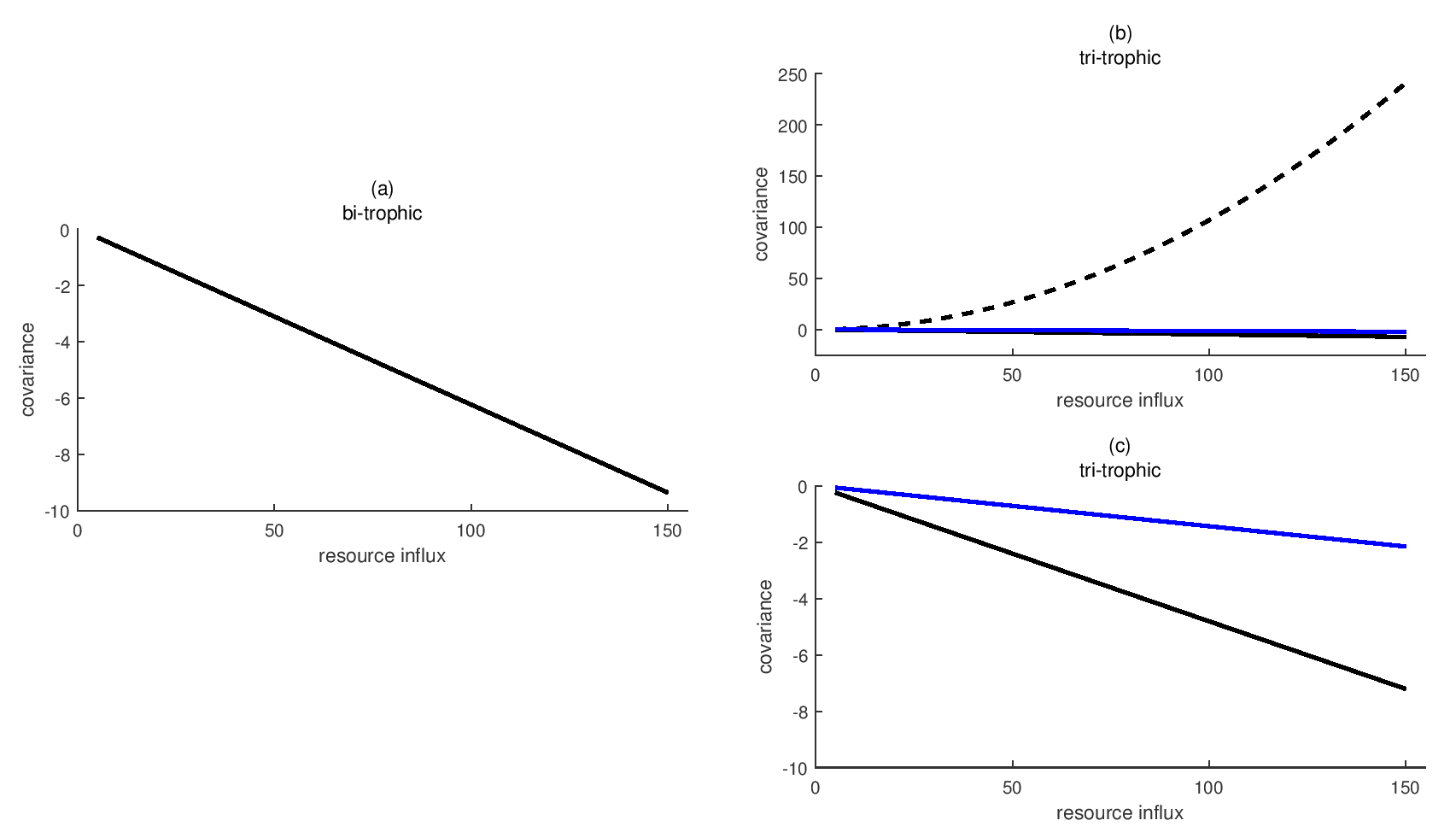


Figure S6. Synchrony (covariance) between species in bi- (a) and tri-trophic (b-c) food chains. Synchrony (covariance) is calculated from the off-diagonals of the stationary covariance matrix. It represents how the equilibrium biomass of one species fluctuates around its equilibrium in response to a stochastic effect in another species. Line color and style indicate the covariance between species $i$ and $j$: $COV_{12}$ (black, solid), $COV_{13}$ (black, dashed), (blue, solid).


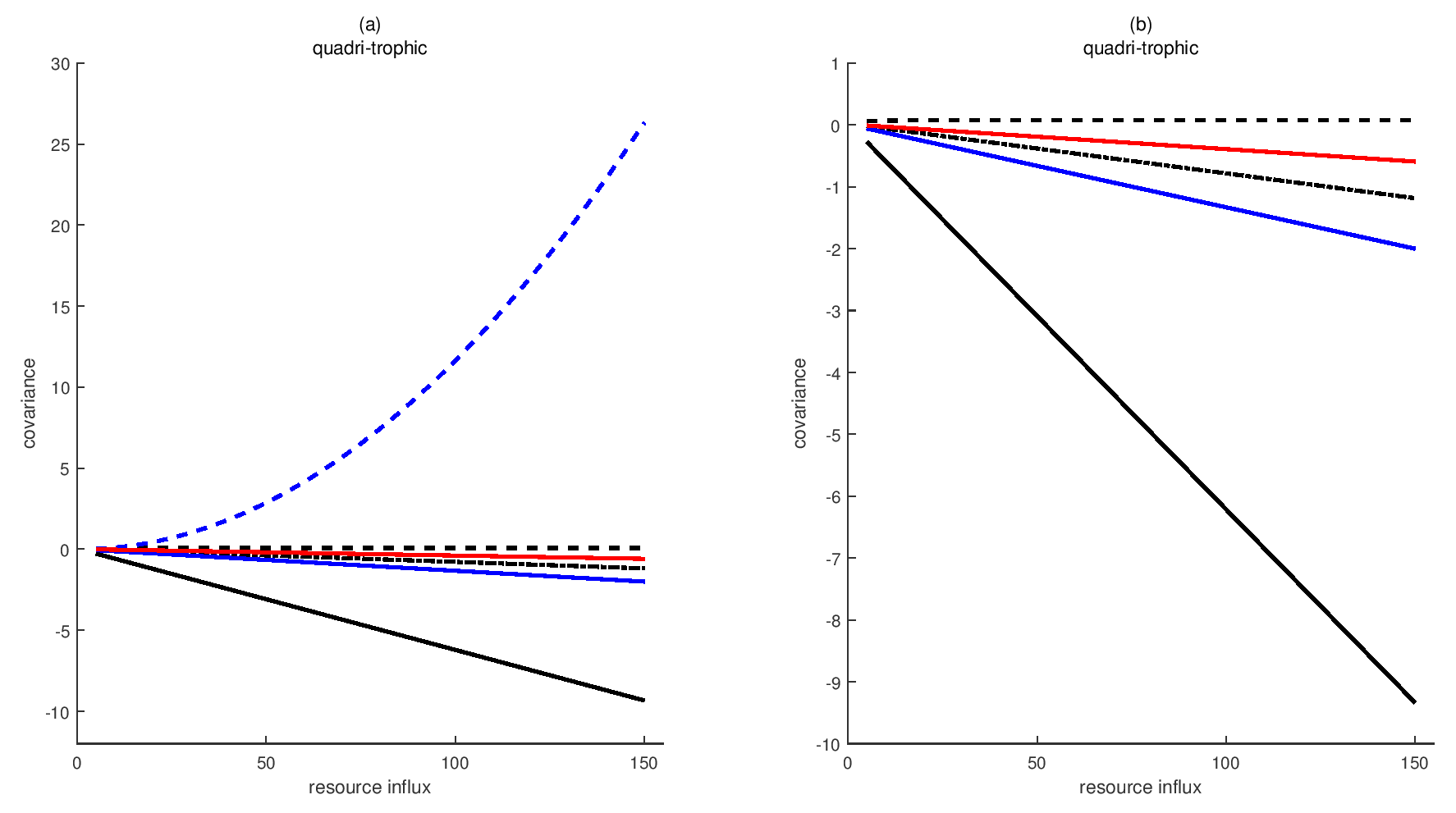


Figure S7. Synchrony between species in a quadri-trophic food chain. Synchrony (covariance) is calculated from the off-diagonals of the stationary covariance matrix. It represents how the equilibrium biomass of one species fluctuates around its equilibrium in response to a stochastic effect in another species. Line color and style indicate the covariance between species $i$ and $j$: $COV_{12}$ (black, solid), $COV_{13}$ (black, dashed), $COV_{14}$ (black, dot-dashed), (blue, solid), (blue, dashed), $COV_{34}$ (red, solid).


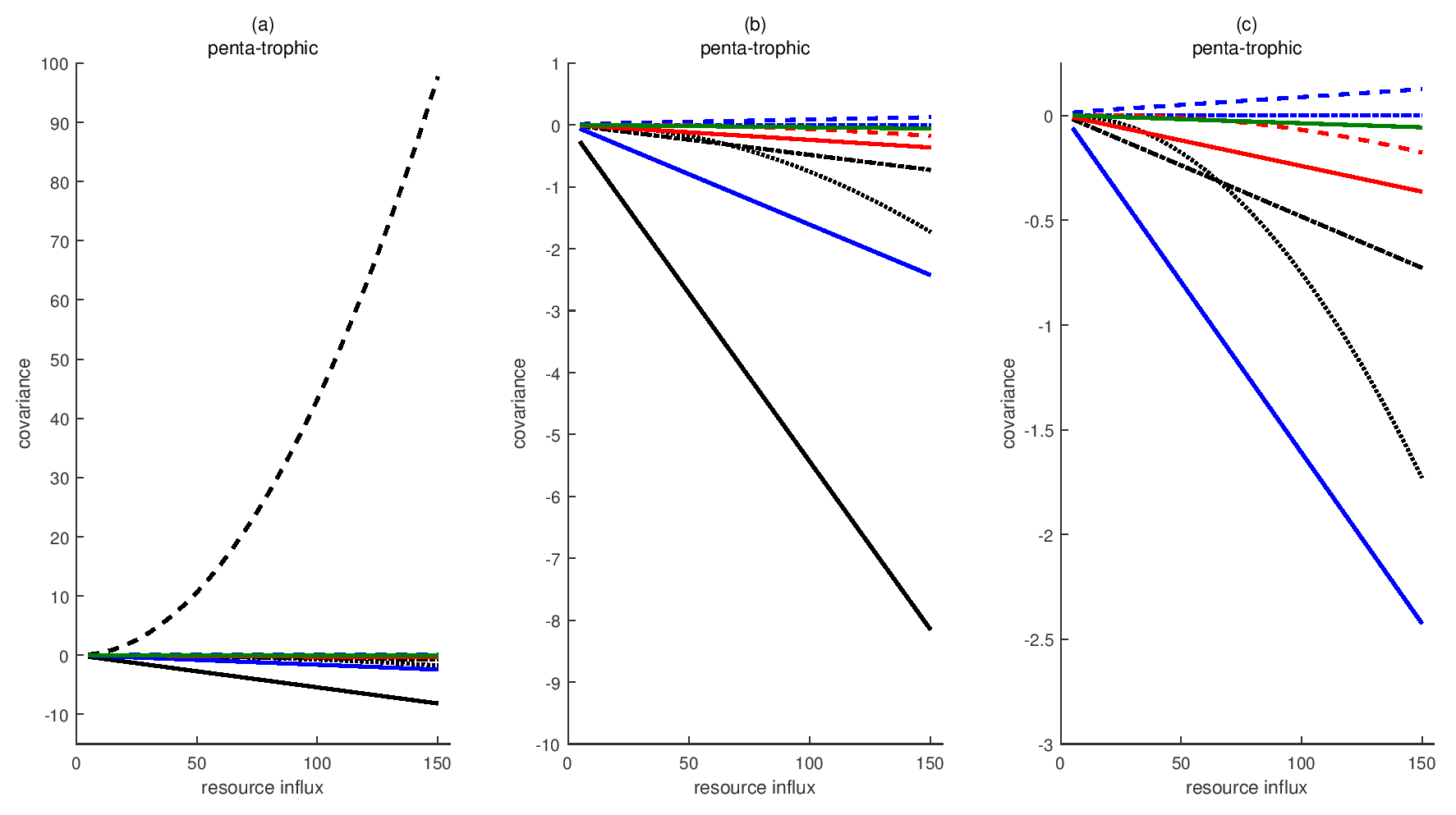


Figure S8. Synchrony between species in a penta-trophic food chain. Synchrony (covariance) is calculated from the off-diagonals of the stationary covariance matrix. It represents how the equilibrium biomass of one species fluctuates around its equilibrium in response to a stochastic effect in another species. Line color and style indicate the covariance between species $i$ and $j$: $COV_{12}$ (black, solid), $COV_{13}$ (black, dashed), $COV_{14}$ (black, dot-dashed), $COV_{15}$ (black, dotted), (blue, solid), (blue, dashed), (blue, dot-dashed), $COV_{34}$ (red, solid), $COV_{35}$ (red, dashed), $COV_{45}$ (green, solid).


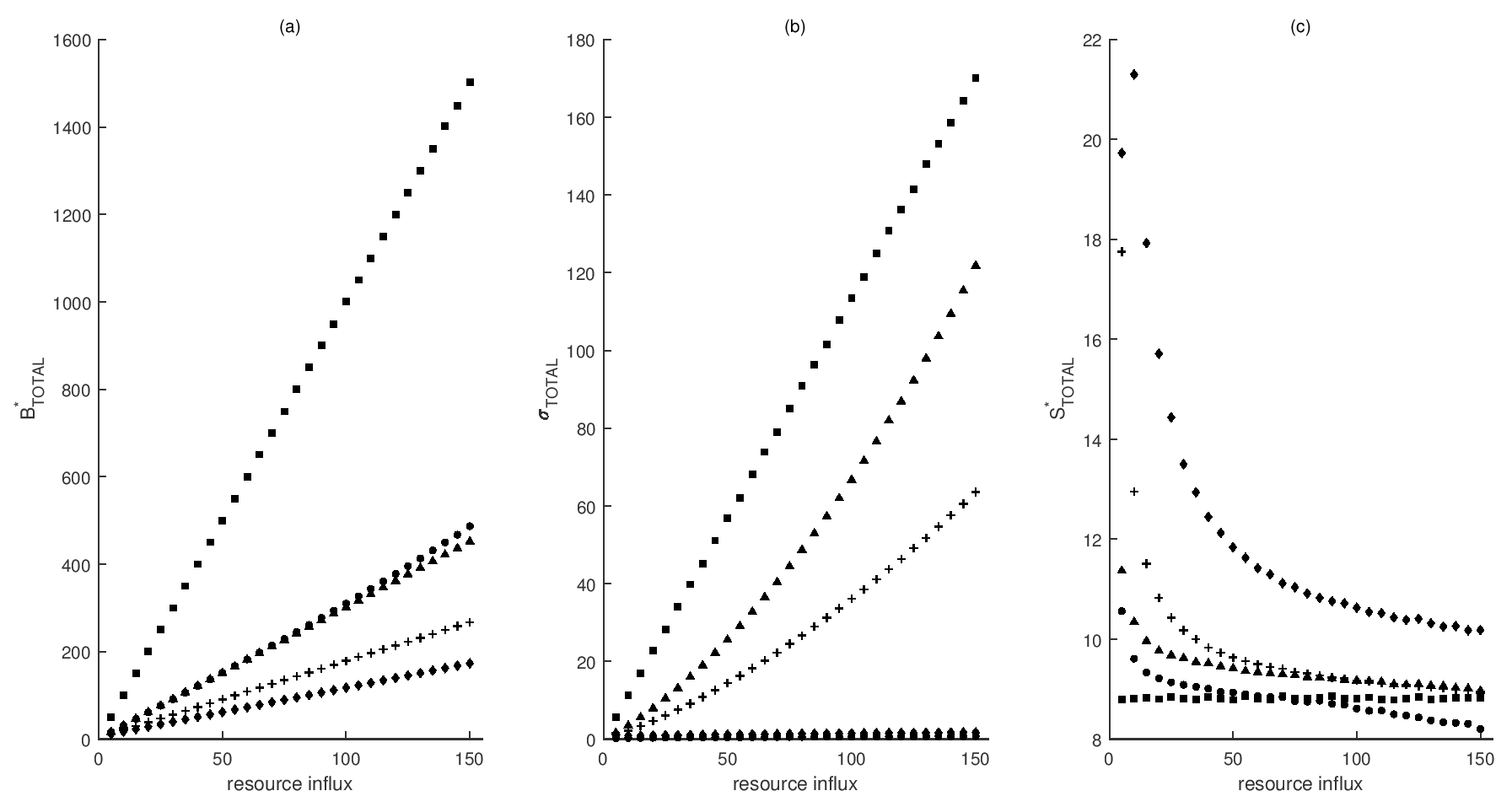


Figure S9. Stability of total biomass across food chains. Panels and marker shape correspond to the equilibrium total biomass (a), temporal standard deviation in total biomass (b), and stability of total biomass (c) across food chains: single species (square), bi-trophic (circle), tri-trophic (triangle), quadri-trophic (diamond), and penta-trophic (plus). Markers are the mean results of 1000 simulations.


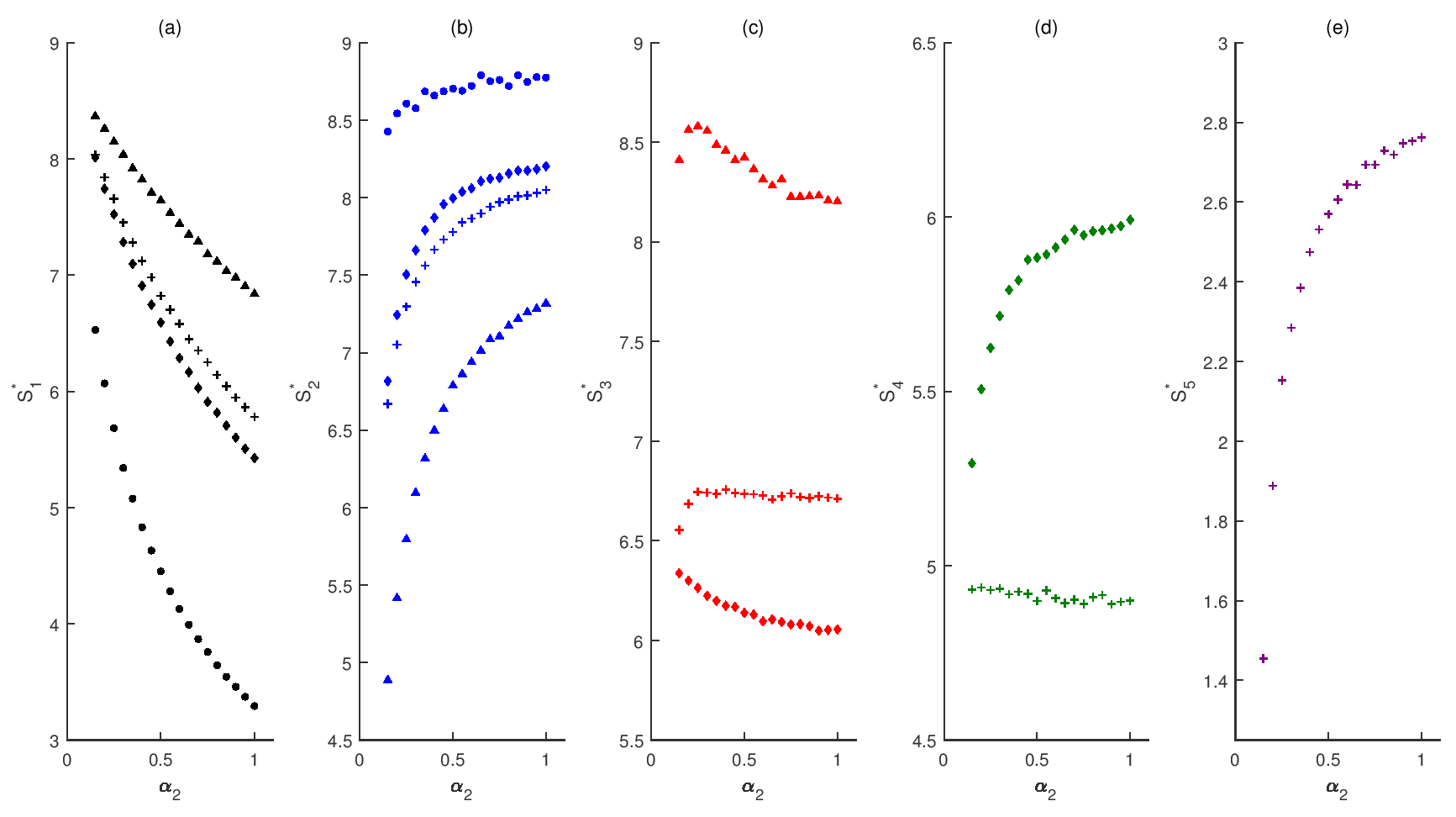


Figure S10. Stability across food chains, varying predation rate of the primary consumer ($\alpha_{2}$). Panels and marker colors correspond to the stability of each species: basal resource (a, black), primary producer (b, blue), primary consumer (c, red), secondary consumer (d, green), and tertiary consumer (e, purple). Marker shape denotes the trophic food chain: bi-trophic (circle), tri-trophic (triangle), quadri-trophic (diamond), and penta-trophic (plus). Markers are the mean results of 1000 simulations.


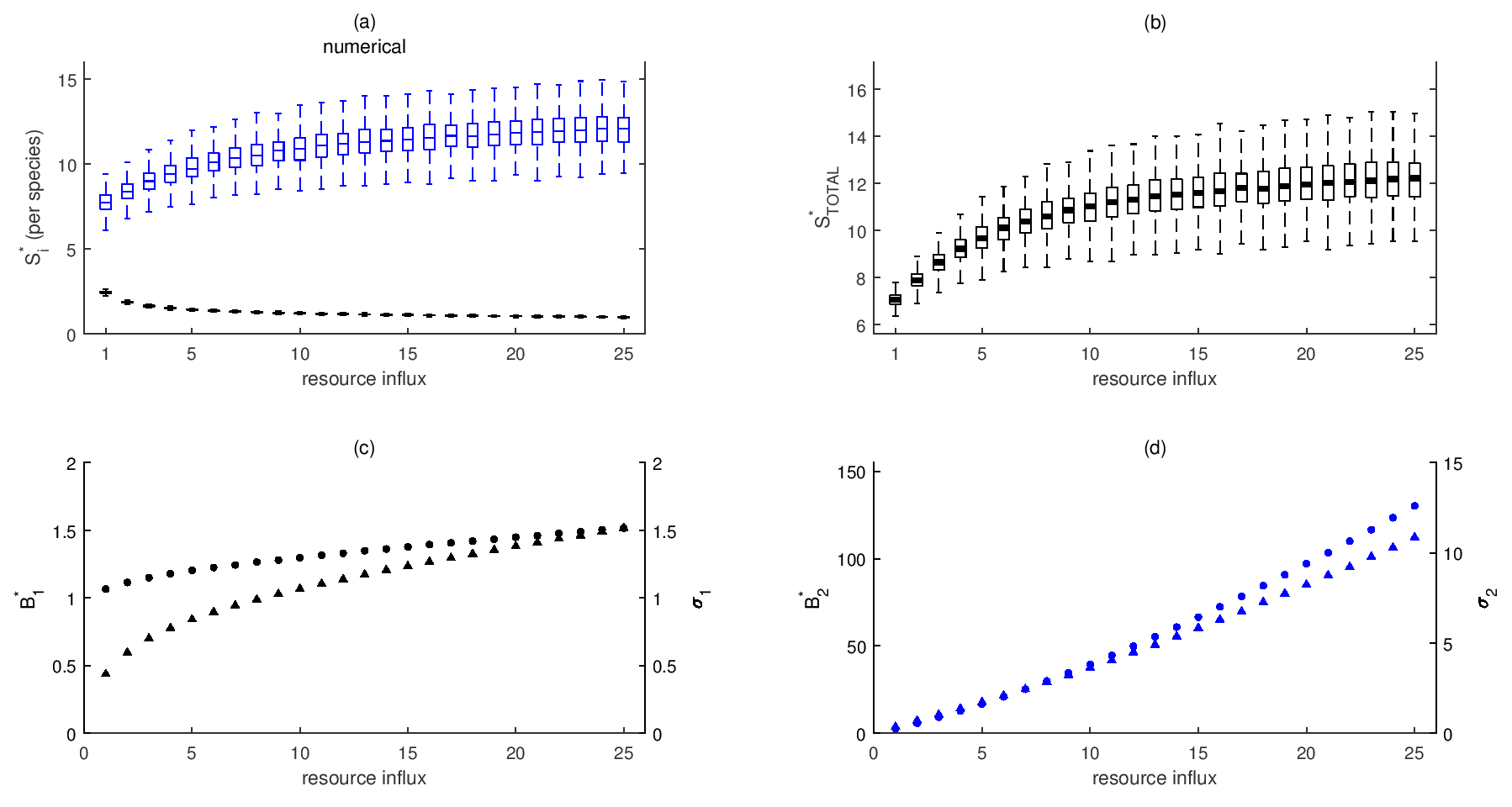


Figure S11. Stability in a *bi-trophic* food chain with a type-II functional response. (a-b) Numerical results for stability per species and the stability of total biomass. Note that stability is presented as *invariability*. Invariability for each species is calculated as the ratio of the standard deviation of biomass and equilibrium biomass (c, d). Color and subscript indicate species: basal resource (black, 1) and primary producer (blue, 2). In (a, b) solid bars indicate the mean values of 1000 simulations. Boxes represent the 25 and 75 percentiles of simulation results. Whiskers correspond to approximately 2.5 standard deviations from the mean. In (c, d) marker shape denotes equilibrium biomass (circle) and standard deviations of biomass (triangle). Markers are the mean value of the simulation results. Primary producer max feeding rate and half-saturation constant are given by $\kappa_{2}=1$ and $D_{2}=2$ respectively. Due to the sensitivity of the simulations to stochasticity, the variances of stochasticity are $\zeta_{I}=0.25$ and $\zeta_{i}=0.000625$.


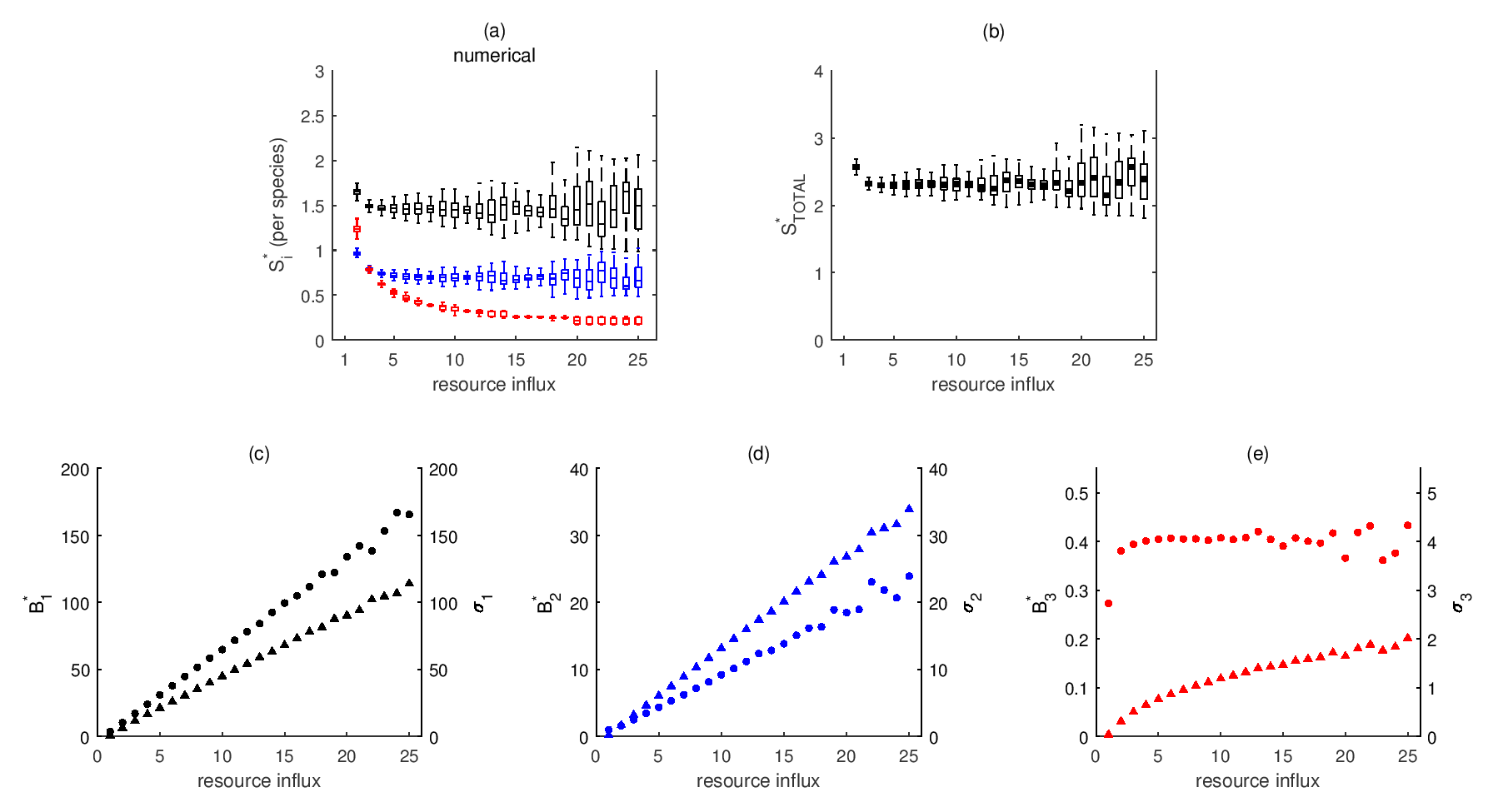


Figure S12. Stability in a *tri-trophic* food chain with a type-II functional response. (a-b) Numerical results for stability per species and the stability of total biomass. Note that stability is presented as *invariability*. Invariability for each species is calculated as the ratio of the standard deviation of biomass and equilibrium biomass (c-e). Color and subscript indicate species: basal resource (black, 1), primary producer (blue, 2), and primary consumer (red, 3). In (a, b) solid bars indicate the mean values of 1000 simulations. Boxes represent the 25 and 75 percentiles of simulation results. Whiskers correspond to approximately 2.5 standard deviations from the mean. In (c-e) marker shape denotes equilibrium biomass (circle) and standard deviations of biomass (triangle). Markers are the mean value of the simulation results. Primary producer and primary consumer max feeding rates and half-saturation constants are given by $\kappa_{i}=1$ and $D_{i}=2$ respectively. Due to the sensitivity of the simulations to stochasticity, we restrict our analysis to three trophic levels and the variances of stochasticity are $\zeta_{I}=0.25$ and $\zeta_{i}=0.000625$.


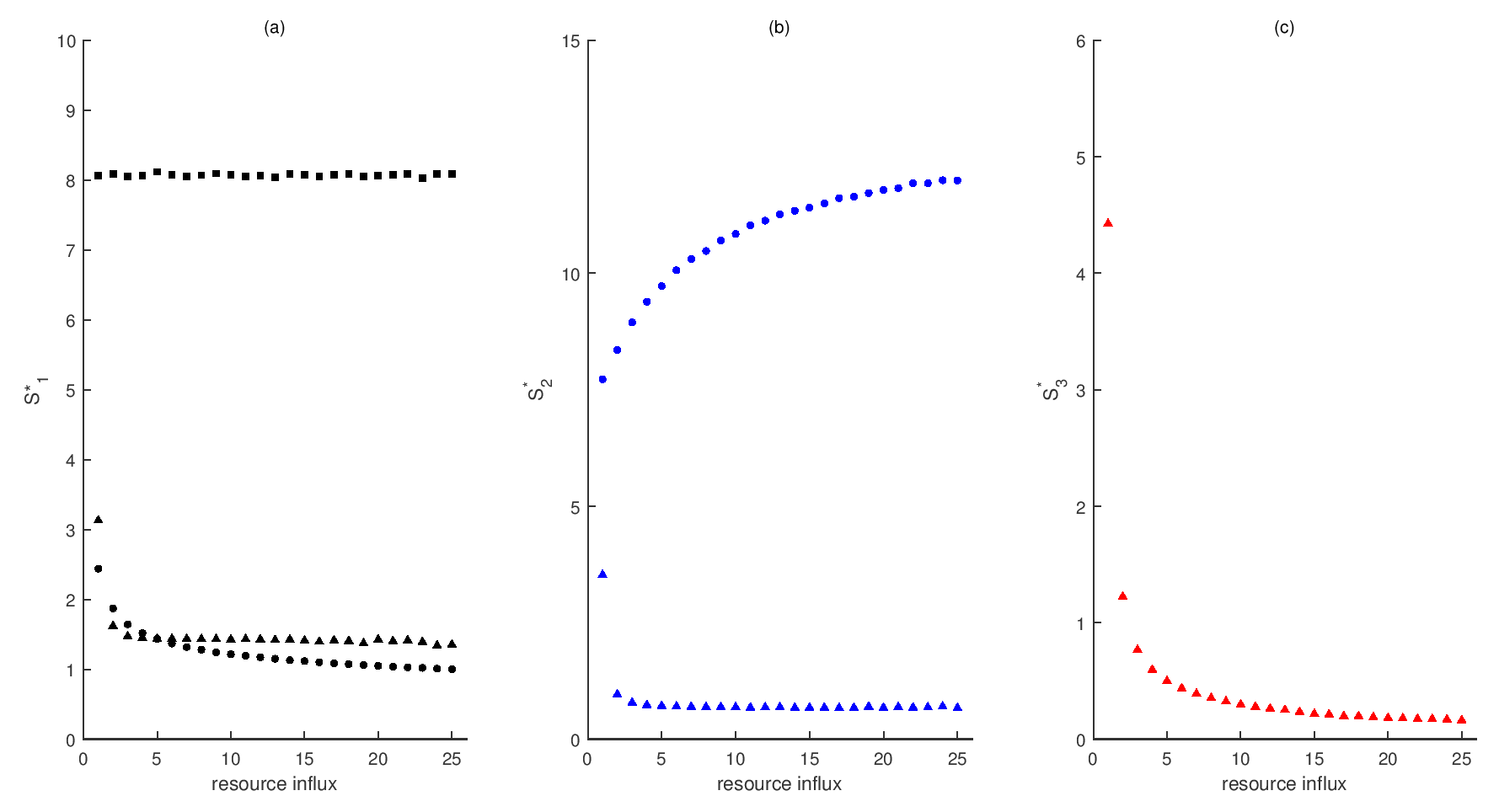


Figure S13. Stability across food chains with a type-II functional response. Note that stability is presented as *invariability*, calculated as the ratio of the temporal mean and standard deviation of biomass. Panels and marker colors correspond to the equilibrium biomass of species: basal resource (a, black), primary producer (b, blue) and primary consumer (c, red). Marker shape denotes the trophic food chain: single species (square), bi-trophic (circle), and tri-trophic (triangle). Markers are the mean results of 1000 simulations. Primary producer and primary consumer max feeding rates and half-saturation constants are given by $\kappa_{i}=1$ and $D_{i}=2$ respectively. Due to the sensitivity of the simulations to stochasticity, we restrict our analysis to three trophic levels and the variances of stochasticity are $\zeta_{I}=0.25$ and $\zeta_{i}=0.000625$.


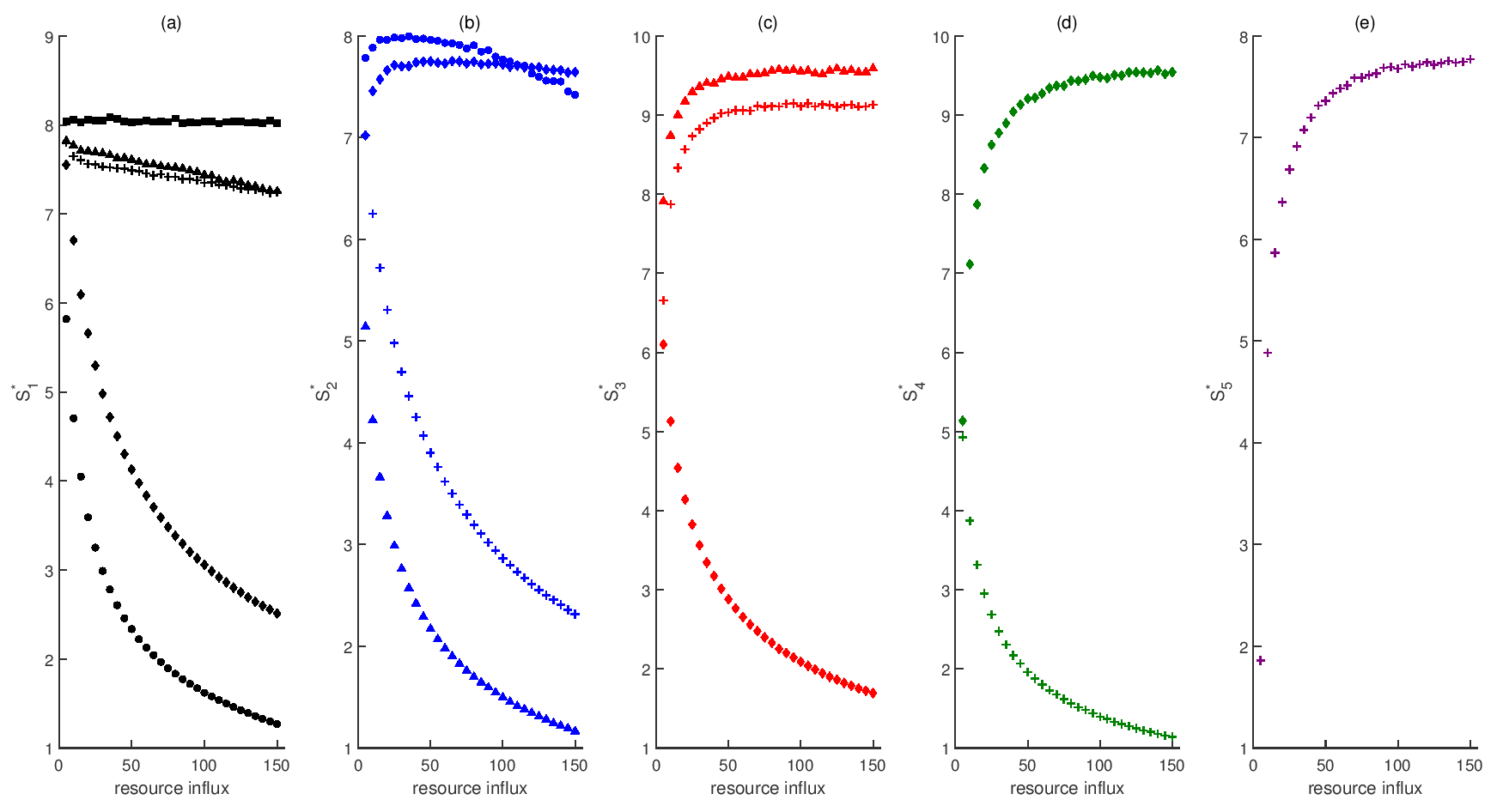


Figure S14. Stability across food chains with a higher rate of stochasticity in the top predator. Panels and marker colors correspond to the stability of species: basal resource (a, black), primary producer (b, blue), primary consumer (c, red), secondary consumer (d, green), and tertiary consumer (e, purple). Marker shape denotes the trophic food chain: single species (square), bi-trophic (circle), tri-trophic (triangle), quadri-trophic (diamond), and penta-trophic (plus). Markers are the mean results of 1000 simulations. Variances of white noise for the top predators are $\zeta_{i}=0.003025$. The variances of all other species are held constant at their baseline values.


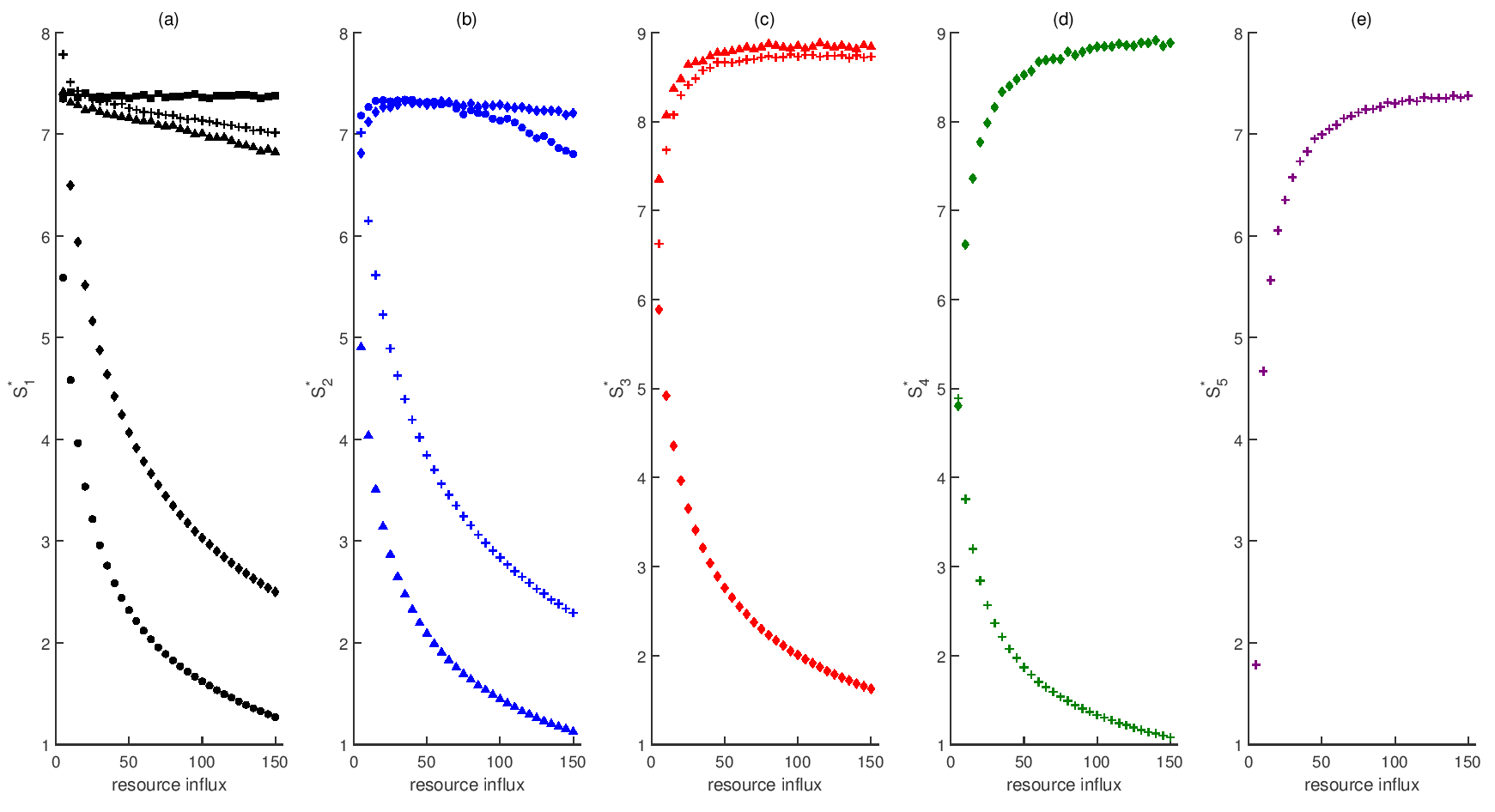


Figure S15. Stability across food chains with a higher rate of stochasticity in the top predator. Panels and marker colors correspond to the stability of species: basal resource (a, black), primary producer (b, blue), primary consumer (c, red), secondary consumer (d, green), and tertiary consumer (e, purple). Marker shape denotes the trophic food chain: single species (square), bi-trophic (circle), tri-trophic (triangle), quadri-trophic (diamond), and penta-trophic (plus). Markers are the mean results of 1000 simulations. Variances of white noise for the top predators are $\zeta_{i}=0.0036$. The variances of all other species are held constant at their baseline values.


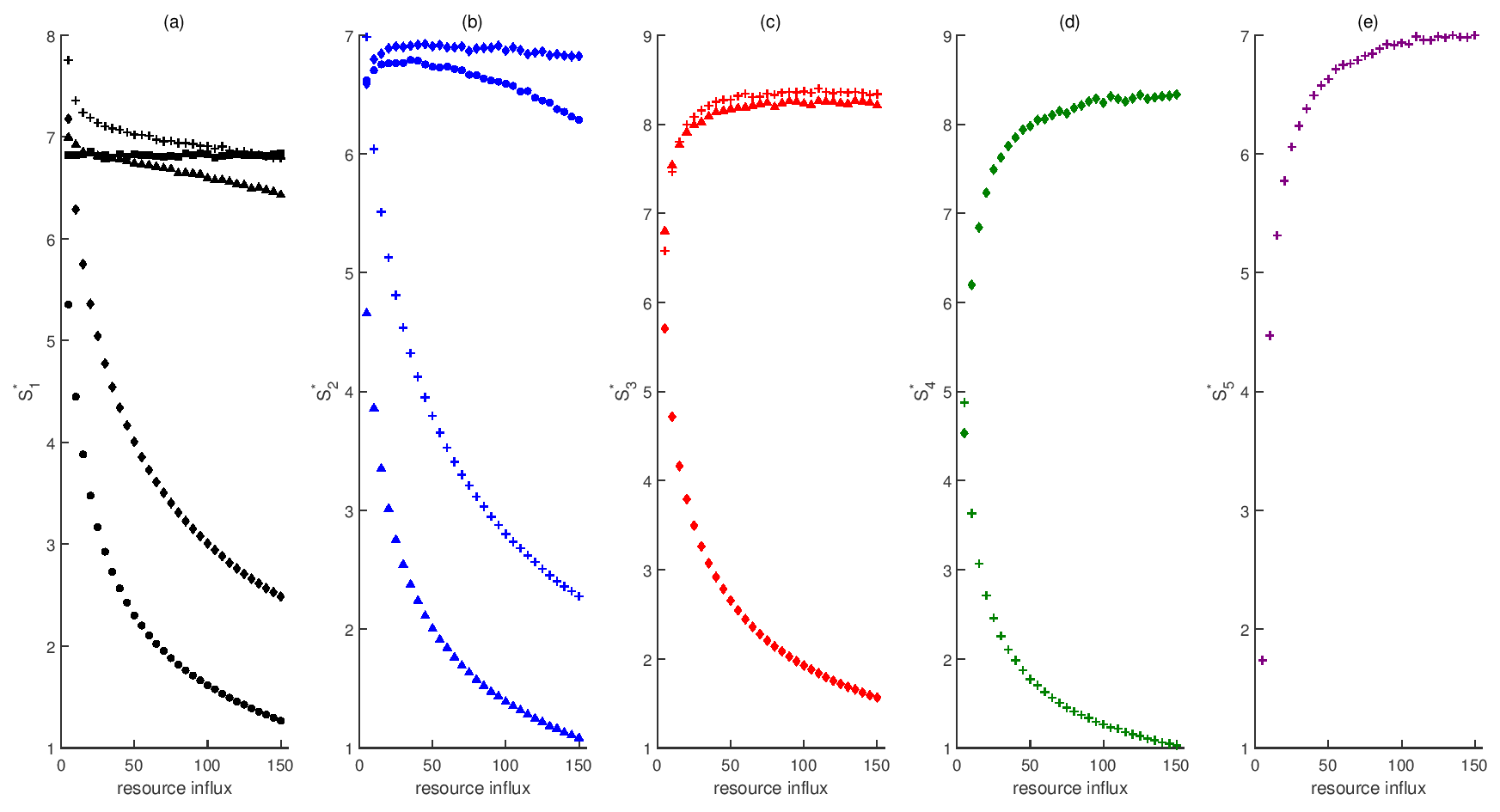


Figure S16. Stability across food chains with a higher rate of stochasticity in the top predator. Panels and marker colors correspond to the stability of species: basal resource (a, black), primary producer (b, blue), primary consumer (c, red), secondary consumer (d, green), and tertiary consumer (e, purple). Marker shape denotes the trophic food chain: single species (square), bi-trophic (circle), tri-trophic (triangle), quadri-trophic (diamond), and penta-trophic (plus). Markers are the mean results of 1000 simulations. Variances of white noise for the top predators are $\zeta_{i}=0.004225$. The variances of all other species are held constant at their baseline values.


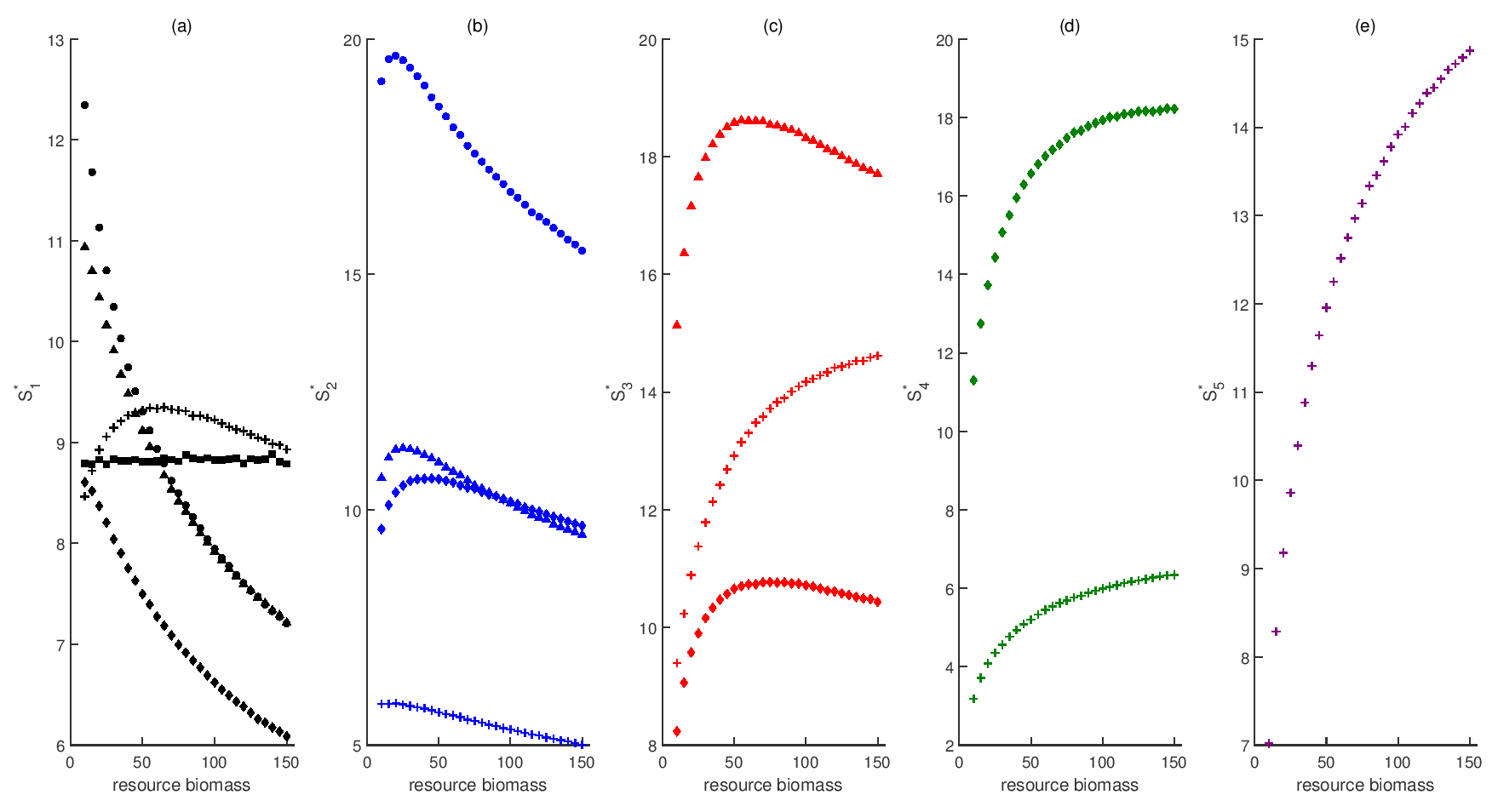


Figure S17. Stability across food chains with density-dependent mortality in the top predator. Panels and marker colors correspond to the stability of species: basal resource (a, black), primary producer (b, blue), primary consumer (c, red), secondary consumer (d, green), and tertiary consumer (e, purple). Marker shape denotes the trophic food chain: single species (square), bi-trophic (circle), tri-trophic (triangle), quadri-trophic (diamond), and penta-trophic (plus). Markers are the mean results of 1000 simulations. The equations for each species are identical to (0.1) except that in each food chain mortality in the top predator is modeled as a quadratic.


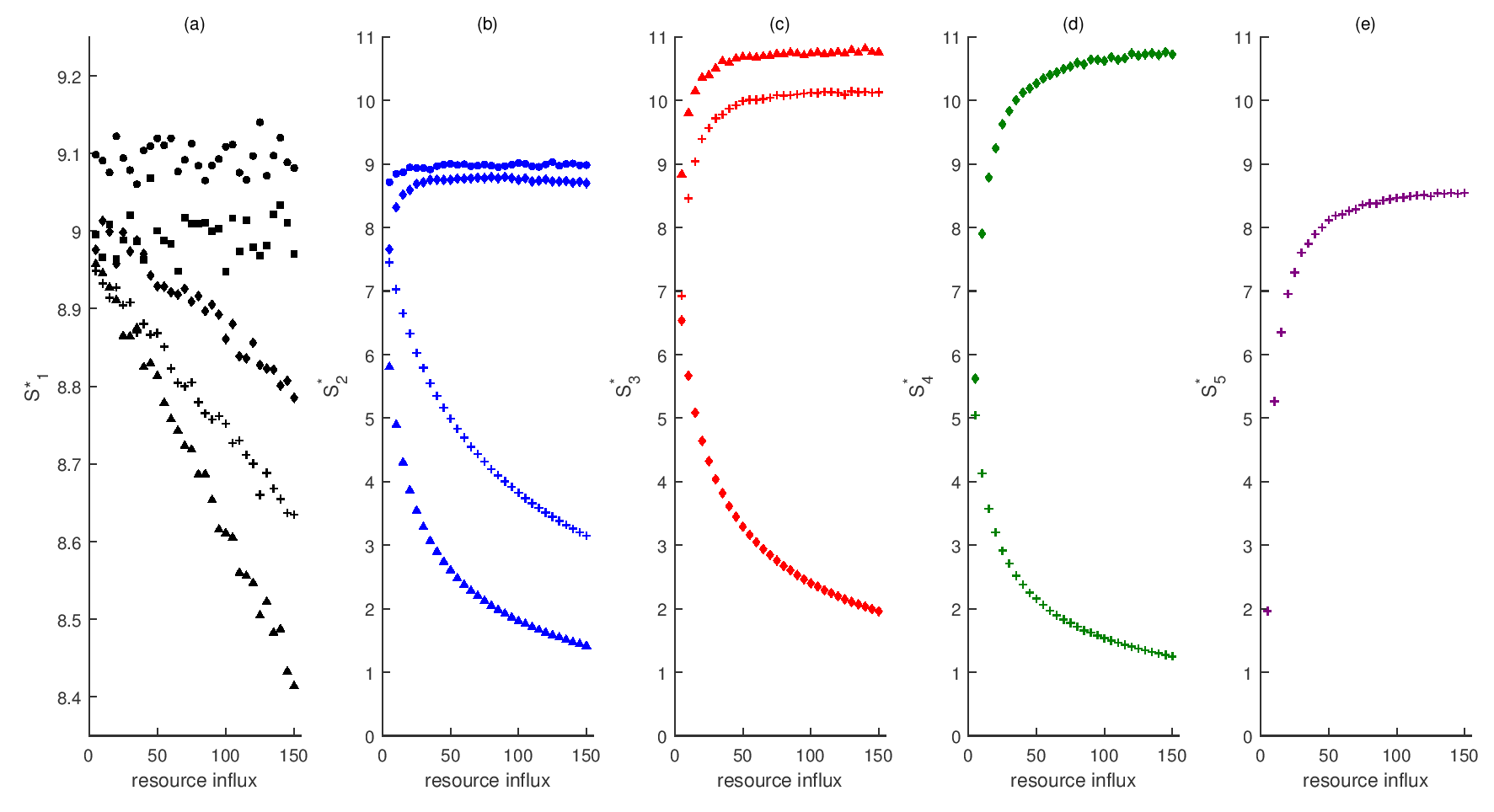


Figure S18. Stability across food chains in the absence of stochasticity in the baseline rate of resource influx. Panels and marker colors correspond to the equilibrium biomass of species: basal resource (a, black), primary producer (b, blue), primary consumer (c, red), secondary consumer (d, green), and tertiary consumer (e, purple). Marker shape denotes the trophic food chain: single species (square), bi-trophic (circle), tri-trophic (triangle), quadri-trophic (diamond), and penta-trophic (plus). Markers are the mean results of 1000 simulations.

SUPPLEMENTAL MATERIAL C. SOURCE CODE FOR ANALYTICAL SOLUTION

(* We build the components of the analytical solution with each piece of the solution treated completely separately, and then solve for the Lyapunov via a loop. By substituting parameter values at each step, it greatly simplifies the solution process. *)

(* Code was created in Mathematica 10.2 *)

(* It is very important to realize that Mathematica is sensitive to:

a) data being in the kernal

b) consistency between array and matrix notation (for example, Lyapunov will not solve if one matrix is in array and the other in vector) *)

(* set of differential equations (food chain, no dispersal) *)

R'[t]=F-l*R[t]-H[t]*f_H_[t,R] ;(* basal resource *)

H'[t]=β_H_*H[t]*f_H_[t,R]-P1[t]*f_P1_[t,H]-H[t]*m_H_ ;(* herbivore *)

P1'[t]=β_P1_*P1[t]*f_P1_[t,H]-P2[t]*f_P2_[t,P1]-P1[t]*m_P1_ ;(* primary predator *)

P2'[t]=β_P2_*P2[t]*f_P2_[t,P1]-T1[t]*f_T1_[t,P2]-P2[t]*m_P2_ ;(* secondary predator *)

T1'[t]=β_P2_*T1[t]*f_T1_[t,P2]-T1[t]*m_T1_; (* tertiary predator *)

(* predator response functions *)

f_H_[t,R]=α_H_*R[t];

f_P1_[t,H]=α_P1_*H[t];

f_P2_[t,P1]=α_P2_*P1[t];

f_T1_[t,P2]=α_T1_*P2[t];

(* define the Jacobian *)

J={{a11,a12,a13,a14,a15},{a21,a22,a23,a24,a25},{a31,a32,a33,a34,a35},{a41,a42,a43,a44,a45},{a51,a52,a53,a54,a55}};

a11=D[R'[t],R[t]];a12=D[R'[t],H[t]];a13=D[R'[t],P1[t]];a14=D[R'[t],P2[t]];a15=D[R'[t],T1[t]];

a21=D[H'[t],R[t]];a22=D[H'[t],H[t]];a23=D[H'[t],P1[t]];a24=D[H'[t],P2[t]];a25=D[H'[t],T1[t]];

a31=D[P1'[t],R[t]];a32=D[P1'[t],H[t]];a33=D[P1'[t],P1[t]];a34=D[P1'[t],P2[t]];a35=D[P1'[t],T1[t]];

a41=D[P2'[t],R[t]];a42=D[P2'[t],H[t]];a43=D[P2'[t],P1[t]];a44=D[P2'[t],P2[t]];a45=D[P2'[t],T1[t]];

a51=D[T1'[t],R[t]];a52=D[T1'[t],H[t]];a53=D[T1'[t],P1[t]];a54=D[T1'[t],P2[t]];a55=D[T1'[t],T1[t]];

(*J//MatrixForm*)

(* solve for the steady states *)

Equil=Solve[{R'[t]==0,H'[t]==0,P1'[t]==0,P2'[t]==0,T1'[t]==0},{R[t],H[t],P1[t],P2[t],T1[t]}];

(* now we will solve for the effect of environmental noise on the dynamics around equilibrium *)

ENVnoise=DiagonalMatrix[{t_F_,t_1_,t_2_,t_3_,t_4_,t_5_}] (* basic white noise, affecting each species separately *)

EQUILmat={{F,R*,0,0,0,0},{0,0,H*,0,0,0},{0,0,0,P1*,0,0},{0,0,0,0,P2*,0},{0,0,0,0,0,T1*}}(* matrix of equilibrium biomass of the states *)

ENVeffect=%.%%.Transpose[%]/.{t_1_->0.05^2,t_2_->0.05^2,t_3_->0.05^2,t_4_->0.05^2,t_5_->0.05^2,t_F_->0.05^2} (*full variance of environmental stochasticity *)

(* calculating invariability via the Lyapunov *)

data={}; (* empty matrix to store the data *)

For[j=5,j<=150,j+=5, (* cycles through rates of resource influx *)

{

(* analytical equations for equilibria, substituting in parameters *)

R0=R[t]/.Equil[[8]]/.{F->j,l->0.1,α_H_->0.2,α_P1_->0.2,α_P2_->0.2,α_T1_->0.5,β_H_->0.3,β_P1_->0.3,β_P2_->0.4,β_T1_->0.4,m_H_->0.1,m_P1_->0.1,m_P2_->0.1,m_T1_->0.1},

H0=H[t]/.Equil[[8]]/.{F->j,l->0.1,α_H_->0.2,α_P1_->0.2,α_P2_->0.2,α_T1_->0.5,β_H_->0.3,β_P1_->0.3,β_P2_->0.4,β_T1_->0.4,m_H_->0.1,m_P1_->0.1,m_P2_->0.1,m_T1_->0.1},

P10=P1[t]/.Equil[[8]]/.{F->j,l->0.1,α_H_->0.2,α_P1_->0.2,α_P2_->0.2,α_T1_->0.5,β_H_->0.3,β_P1_->0.3,β_P2_->0.4,β_T1_->0.4,m_H_->0.1,m_P1_->0.1,m_P2_->0.1,m_T1_->0.1},

P20=P2[t]/.Equil[[8]]/.{F->j,l->0.1,α_H_->0.2,α_P1_->0.2,α_P2_->0.2,α_T1_->0.5,β_H_->0.3,β_P1_->0.3,β_P2_->0.4,β_T1_->0.4,m_H_->0.1,m_P1_->0.1,m_P2_->0.1,m_T1_->0.1},

T10=T1[t]/.Equil[[8]]/.{F->j,l->0.1,α_H_->0.2,α_P1_->0.2,α_P2_->0.2,α_T1_->0.5,β_H_->0.3,β_P1_->0.3,β_P2_->0.4,β_T1_->0.4,m_H_->0.1,m_P1_->0.1,m_P2_->0.1,m_T1_->0.1},

(*Print[{j,R0,H0,P10,P20,T10}],*)

(* environmental effect *)

ELyap=ENVeffect/.{F->j,R*->R0,H*->H0,P1*->P10,P2*->P20,T1*->T10}//Chop//Simplify,

(*Print[ELyap],*)

(* Jacobian evaluated at equilibrium *)

JLyap=J/.{R[t]->R0,H[t]->H0,P1[t]->P10,P2[t]->P20,T1[t]->T10,F->j,l->0.1,α_H_->0.2,α_P1_->0.2,α_P2_->0.2,α_T1_->0.5,β_H_->0.3,β_P1_->0.3,β_P2_->0.4,β_T1_->0.4,m_H_->0.1,m_P1_->0.1,m_P2_->0.1,m_T1_->0.1}//Chop//Simplify,

(*Print[JLyap],*)

(* solve the Lyapunov equation *)

LY=LyapunovSolve[-JLyap,ELyap]//Chop,

(*Print[LY],*)

(* constructs the final dataset of variability *)

data=AppendTo[data,{

j, (* rate of resource influx *)

(* variability per species *)

Sqrt[LY[[1,1]]]/R0,

Sqrt[LY[[2,2]]]/H0,

Sqrt[LY[[3,3]]]/P10,

Sqrt[LY[[4,4]]]/P20,

Sqrt[LY[[5,5]]]/T10,

(* average variability across all species *)

Mean[{Sqrt[LY[[1,1]]]/R0,Sqrt[LY[[2,2]]]/H0,Sqrt[LY[[3,3]]]/P10,Sqrt[LY[[4,4]]]/P20,Sqrt[LY[[5,5]]]/T10}],

(* gamma variability *)

Sqrt[Total[Total[LY]]]/Total[{R0,H0,P10,P20,T10}],

(* synchrony *)

LY[[2,1]],

LY[[3,1]],

LY[[3,2]],

LY[[4,1]],

LY[[4,2]],

LY[[4,3]],

LY[[5,1]],

LY[[5,2]],

LY[[5,3]],

LY[[5,4]]

}

] (* End of AppendTo *)

}

] (* End of 'for' loop *)

(* Note that to calculate invariability, take the inverse of variability *)

Export["data_Lyapunov.xlsx",data]

SUPPLEMENTAL MATERIAL D. SOURCE CODE FOR NUMERICAL SOLUTION

% penta-trophic food chain w/ a type-I/-II functional response (lower-level food chains can be modified from this)

% fundamentally investigate the effects of adding trophic levels on biodiversity and stability

% code was designed in MatLab 2016a

clear

clc

rng(987609) % set random seed

p.MaxTime=5000; % time horizon of simulation

p.dt=0.01; % step size

p.T=floor(p.MaxTime/p.dt); % total number of iterations

p.epsilon=1e-5; % tolerance for equilibrium

%%% model parameters %%%

p.npa=1; % number of patches

p.ntl=5; % number of trophic levels

p.nsim=1000;

p.ITotal=5:5:150; % (5:5:150)

% p.I=5; % basal resource influx rate (10; 2)

p.l=0.1; % basal resource loss rate (0.1; 0.4)

% p.alphaPTotal=0.15:0.05:1;

p.alphaP=0.2; % primary producer feeding rate (0.2; 1)

% p.alphaC1Total=0.05:0.05:1;

p.alphaC1=0.2; % primary consumer feeding rate (0.2; 1)

% p.alphaC2Total=0.15:0.05:1;

p.alphaC2=0.2; % secondary consumer feeding rate (0.2; 1)

% p.alphaC3Total=0.15:0.05:1;

p.alphaC3=0.5; % secondary consumer feeding rate (0.2; 1)

p.betaP=0.3; % primary producer biomass conversion efficiency (0.2; 1; 0.3)

p.betaC1=0.3; % primary consumer biomass conversion efficiency (0.2; 1; 0.3)

p.betaC2=0.4; % secondary consumer biomass conversion efficiency (0.4)

p.betaC3=0.4; % secondary consumer biomass conversion efficiency (0.4)

p.mP=0.1; % primary producer mortality rate

p.mC1=0.1; % primary consumer mortality rate

p.mC2=0.1; % secondary consumer mortality rate

p.mC3=0.1; % secondary consumer mortality rate

% environmental stochasticity

p.mu=0; % mean

p.sigma=1; % standard deviation

p.theta=0.05; % weight of the environmental response (equivalent to ~N(0, theta^2 * sigma)

%%% main iteration %%%

p.InitCond=[5.87 3.33 1.26 0.5 0.0017]; % initial conditions ([6 3.75 1.25 0.5]; [8.33 3.33 1.375 0.5 0.02])

options=optimset('display','off','tolfun',1e-8,'tolx',1e-8); % options for the equilibrium solver

% matrices to store data

resbio=zeros(length(p.ITotal*p.nsim),12);

resvar=zeros(length(p.ITotal*p.nsim),9);

for ctl=1:length(p.ITotal)

p.I=p.ITotal(ctl);

% p.alphaP=p.alphaPTotal(ctl);

% p.alphaC1=p.alphaC1Total(ctl);

% p.alphaC2=p.alphaC2Total(ctl);

% calculates equilibrium values to use as initial conditions

equil=[ ((p.I.*p.alphaC1.*p.alphaC3.*p.betaC1.*p.betaC2) ./ (p.mC3.*p.alphaP.*p.alphaC2 + ... p.alphaC3.*p.betaC2.*(p.mC1.*p.alphaP + p.l.*p.alphaC2.*...

p.betaC1))) ...

((p.mC3.*p.alphaC2 + p.mC1.*p.alphaC3.*p.betaC2) ./ (p.alphaC1.*p.alphaC3.*p.betaC1.*p.betaC2)) ...

((p.I.*p.alphaP.*p.alphaC1.*p.alphaC3.*p.betaP.*p.betaC1.*p.betaC2 - p.mP.*p.mC3.*p.alphaP.*p.alphaC2 - p.mP.*p.mC1.*p.alphaP.*p.alphaC3.*...

p.betaC2 - p.l.*p.mP.*p.alphaC1.*p.alphaC3.*p.betaC1.*p.betaC2) ./ (p.alphaC1.*(p.mC3.*p.alphaP.*p.alphaC2 + p.mC1.*p.alphaP.*p.alphaC3.*...

p.betaC2 + p.l.*p.alphaC1.*p.alphaC3.*p.betaC1.*p.betaC2))) ...

(p.mC3 ./ (p.alphaC3.*p.betaC2)) ...

(p.I.*p.alphaP.*p.alphaC1.*p.alphaC2.*p.alphaC3.*p.betaP.*p.betaC1.*(p.betaC2.^2) - p.mC2.*p.mC3.*p.alphaP.*p.alphaC1.*p.alphaC2 - p.mP.*p.mC3.*...

p.alphaP.*(p.alphaC2.^2).*p.betaC2 - p.mC1.*p.mC2.*p.alphaP.*p.alphaC1.*p.alphaC3.*p.betaC2 - p.l.*p.mC2.*(p.alphaC1.^2).*p.alphaC3.*...

p.betaC1.*p.betaC2 - p.mP.*p.mC1.*p.alphaP.*p.alphaC2.*p.alphaC3.*(p.betaC2.^2) - p.l.*p.mP.*p.alphaC1.*p.alphaC2.*p.alphaC3.*p.betaC1.*...

(p.betaC2.^2)) ./ (p.alphaC1.*p.alphaC3.*(p.mC3.*p.alphaP.*p.alphaC2 + p.mC1.*p.alphaP.*p.alphaC3.*p.betaC2 + p.l.*p.alphaC1.*p.alphaC3.*...

p.betaC1.*p.betaC2))];

p.InitCond=equil;

% solve for the equilibrium (steady state) values using MatLab's solver

% [equil,aux]=fsolve(@(x) model_metacomm_rhs(x,p),p.InitCond,options);

% p.InitCond=equil;

for n=1:p.nsim

% solve the trajectory (ode45)

% options45=optimset('display','off','tolfun',1e-8,'tolx',1e-8); % options for the ODE solver

% [t,x]=ode45(@(t,x) model_metacomm_rhs(x,p),0:p.MaxTime,p.InitCond,options45); % solve the trajectory

% figure(1);plot(t,x);

% solves the system of ODEs using ode15s (ode15s)

% options15s=odeset('reltol',1e-8,'abstol',1e-8,'jacobian',@(t,x) model_metacomm_jac(x,p)); % resets the options

to include the Jacobian (ode15s)

% [tt,xx]=ode15s(@(t,x) model_metacomm_rhs(x,p),[0 p.MaxTime],p.InitCond,options15s);

% figure(2);plot(tt,xx);

% solves the system of ODEs using a 1st-order Euler approximation

[x,xequil,xsum]=discrete_sys(p);

% plots trajectory

% figure(3);

% hold on;

% plot(1:p.T,x(:,1));plot(1:p.T,x(:,2));plot(1:p.T,x(:,3));plot(1:p.T,x(:,4));plot(1:p.T,x(:,5));

% hold off;

% calculate total biomass and stability

resbio(ctl*p.nsim-p.nsim+n,1:12)=[p.ITotal(ctl) mean(xequil(:,1)) mean(xequil(:,2)) mean(xequil(:,3)) ...

mean(xequil(:,4)) mean(xequil(:,5)) mean(xsum) equil'];

resvar(ctl*p.nsim-p.nsim+n,1:9)=[p.ITotal(ctl) std(xequil)./mean(xequil) mean(std(xequil)./mean(xequil)) ...

std(xsum)/mean(xsum) (std(xsum)/mean(xsum))/(mean(std(xequil)./mean(xequil)))];

end

% display notification if at a specified number of simulations

% if rem(p.ITotal(ctl),25)==0

% display(p.ITotal(ctl))

% end

% p.alphaC1Total(ctl)

end

% save the data

% save('RPC1C2C3_I_theta=0.05_numerical.mat','resbio','resvar');

% save('RPC1C2C3_alphaP_sigma=1_theta=0.05_numerical.mat','resbio','resvar');

% Note that the data in 'resvar' is variability. In order to calculate invariability, you need to take the inverse, e.g.

% 1./resvar

% discrete time Euler approximation

function [x,xequil,xsum]=discrete_sys(p)

% matrxi to store data

x=zeros(p.T,p.npa*p.ntl);

x(1,:)=p.InitCond;

% environmental stochasticity

eta=normrnd(p.mu,p.sigma,p.T,p.ntl);

etaI=normrnd(0,0.05,p.T,1);

% the main iteration

for t=1:p.T-1

% basal resource

x(t+1,1)=x(t,1) + p.dt.*(p.I) + sqrt(p.dt).*( etaI(t).*p.I ) + p.dt.*( -p.l.*x(t,1) - p.alphaP.*x(t,1).*x(t,2) ) + ...

sqrt(p.dt).*( p.theta.*eta(t,1).*x(t,1) );

x(t+1,1)=double(x(t+1,1)>=0).*x(t+1,1); % extinction threshold

% primary producer

x(t+1,2)=x(t,2) + p.dt.*( p.alphaP.*p.betaP.*x(t,1).*x(t,2) - p.alphaC1.*x(t,2).*x(t,3) - x(t,2).*p.mP ) + ...

sqrt(p.dt).*( p.theta.*eta(t,2).*x(t,2) );

x(t+1,2)=double(x(t+1,2)>=0).*x(t+1,2); % extinction threshold

% primary consumer

x(t+1,3)=x(t,3) + p.dt.*( p.alphaC1.*p.betaC1.*x(t,2).*x(t,3) - p.alphaC2.*x(t,3).*x(t,4) - x(t,3).*p.mC1 ) + ...

sqrt(p.dt).*( p.theta.*eta(t,3).*x(t,3) );

x(t+1,3)=double(x(t+1,3)>=0).*x(t+1,3); % extinction threshold

% secondary consumer

x(t+1,4)=x(t,4) + p.dt.*( p.alphaC2.*p.betaC2.*x(t,3).*x(t,4) - p.alphaC3.*x(t,4).*x(t,5) - x(t,4).*p.mC1 ) + ...

sqrt(p.dt).*( p.theta.*eta(t,4).*x(t,4) );

x(t+1,4)=double(x(t+1,4)>=0).*x(t+1,4); % extinction threshold

% tertiary consumer

x(t+1,5)=x(t,5) + p.dt.*( p.alphaC3.*p.betaC3.*x(t,4).*x(t,5) - x(t,5).*p.mC3 ) + sqrt(p.dt).* ...

(p.theta.*eta(t,5).*x(t,5) );

x(t+1,5)=double(x(t+1,5)>=0).*x(t+1,5); % extinction threshold

end

xequil=x((p.MaxTime-1000)/p.dt:1/p.dt:end,:); % construct dataset of system at equilibrium

xsum=sum(xequil,2);

end
